# A 0.002 cm^−1^-Accurate PES for ^14^N_2_^16^O

**DOI:** 10.3390/molecules31111793

**Published:** 2026-05-23

**Authors:** Xinchuan Huang, David W. Schwenke

**Affiliations:** 1 MS 245-6, Astrophysics Branch, NASA Ames Research Center, Moffett Field, CA 94035, USA; 2 SETI Institute, 339 Bernardo Avenue, Suite 200, Mountain View, CA 94043, USA; 3 MS 258-2, NAS Facility, NASA Ames Research Center, Moffett Field, CA 94035, USA; david.schwenke@gmail.com

**Keywords:** potential energy surface, empirical refinement, line list, IR spectroscopy, nitrous oxide, N_2_O, isotopologue, astrochemistry, computational spectroscopy

## Abstract

High-accuracy potential energy surface (PES) and rovibrational energy levels are essential for computational IR line lists used in (exo)planetary atmospheric spectroscopic analysis and modeling. We present a new ^14^N_2_^16^O PES refinement achieving 0.001–0.002 cm^−1^ statistical accuracy for *E*_vib_ ≤ 7000 cm^−1^ and *J*_max_ = 88–100, relative to *complete* experiment-based rovibrational energy levels in RITZ, MARVEL, HITRAN2020, and NOSL-296 datasets. Building upon the high-quality ab initio Comp-I PES, the resulting D2n (and D2nB) PES outperform the Ames B1b PES, the UCL TYM PES, and the UCL 2025 PES series in both energy-resolved and *J*-resolved comparisons, exhibiting the smallest mean residuals and scatter below *E*_vib_ = 8000 cm^−1^, as well as the highest fractions of |δ| < 0.0010 cm^−1^ and |δ| < 0.0005 cm^−1^. Robust analysis identified only seven outliers among the UCL-2025 reference level set; all remaining levels are retained to ensure resilient statistics. The D2n PES also shows stable IR intensities with the G10K dipole moment surface and reasonably consistent isotopologue accuracy. Analysis of *J*-resolved σ_rms_ highlights the critical role of reference-dataset accuracy and *internal consistency*. We discuss factors enabling (sub-)0.002 cm^−1^ accuracy and prospects for extending similar accuracy to higher energies, additional isotopologues, and other molecules.

## 1. Introduction

A molecular potential energy surface (PES) provides a quantitative description of how energy curvature and anharmonicity vary with nuclei motion, thereby linking microscopic molecular forces to macroscopic observables. The accuracy of a PES is fundamental to reliable modeling, interpretation, and prediction in a wide range of research areas, including vibrational dynamics, collisional dynamics, line broadening, and pressure-shift coefficients, etc. In theoretical rotational–vibrational spectroscopy and in the construction of microwave (MW) and infrared (IR) line lists, the PES accuracy largely determines or directly limits the accuracy of computed rovibrational energy levels and transition frequencies, and strongly affects the fidelity of line intensity predictions. Deficiencies in a PES may lead to measurable deviations in calculated (ro)vibrational energy levels and line positions, then propagate into the associated wavefunctions, and ultimately into the MW/IR line intensities that can be measured in laboratory. Consequently, the accuracy of PES governs the overall reliability and applicability of the resulting line lists.

For astrophysical applications, high-fidelity line lists and opacity databases are essential to maximizing the scientific return of NASA/ESA missions by enabling robust atmospheric retrievals, reliable opacity calculations, and meaningful comparisons between observations and models, thereby shaping our understanding of planetary and stellar environments, formation and evolution, and even potential habitability. Specifically, accurate infrared line lists are indispensable for the atmospheric spectra interpretation of Solar system planets and moons, exoplanets, and cool dwarfs, where errors in line positions or intensities may degrade inferences about composition, temperature structure, and atmospheric dynamics. Modeling and interpreting astronomical observations from the IR/Vis instruments aboard JWST (NIRSpec, NIRISS, NIRCam, MIRI; up to 0.6 μm) and the future HWO observatory (extending up to 0.4–0.8 μm) need high-quality rovibrational IR/Vis line lists extending beyond 1 μm (or 10,000 cm^−1^). Therefore, the pursuit of more accurate PESs and line lists directly supports the broader scientific effort to characterize planetary atmospheres and to search for life in the universe.

Over the past three decades, semi-empirical molecular IR line lists have been developed at NASA Ames using a “Best Theory + Reliable High-resolution Experiment” (BTRHE) strategy to integrate the strengths of high-quality ab initio theory with experiment-based refinements and validations [1,2]. Similar approaches are widely used in the community, though implementation details can vary among research groups [3,4]. In general, quantum rovibrational variational configuration-interaction (CI) calculations are performed on high quality ab initio PESs calibrated against reliable high-resolution experimental data to minimize the deviations of energy levels and line positions arising from limitations of ab initio methods. Line positions are obtained as differences between computed energy levels, and IR intensities are computed using high-quality ab initio dipole moment surfaces (DMS). This framework enables complete, reliable and internally consistent predictions for hundreds to millions of transitions that have not been measured, or are difficult to measure in lab, e.g., weak bands, minor isotopologue bands, high temperature spectra, and high energy transitions. Experimental-based line positions can further improve the accuracy of computed IR line lists. Because the quality of PES and DMS are equally important for line list developments, it often requires multiple cycles of PES and DMS upgrades to achieve satisfactory improvements for the overall line lists, or for specific IR bands. Historically, however, PES refinements have an accuracy bottleneck of 0.01–0.03 cm^−1^, which has been the typical σ_rms_ reported by Ames, ExoMol, and TheoReTS groups. Beyond astrophysical and atmospheric applications, it is also scientifically valuable to determine the practical limits of PES accuracy, quantify its impact on IR intensity predictions, and assess the quality and convergence of prior calculations performed on less accurate PESs.

Nitrous Oxide (N_2_O) is an important greenhouse molecule in Earth’s atmosphere, and considered a potential biosignature molecule in exoplanetary environments. Our Ames-1 PES refinement (2021) [5] was published in 2023 [6] with 0.02–0.03 cm^−1^ accuracy for reliable HITRAN2020 [7] levels of N_2_O isotopologues. In 2023, both the Ames and UCL/ExoMol groups accomplished new refinements based on the Comp I PES of Schröder et al. (2015) [8]. In 2024, Yurchenko et al. [9] reported the UCL TYM line list with a PES accuracy of σ_rms_ = 0.02 cm^−1^ for 17,532 MARVEL [10,11] levels [12] in the range of 0–14,000 cm^−1^, with bin-resolved accuracies of 0.003, 0.007, 0.01, 0.01, 0.025, 0.06, and 0.04 cm^−1^ for seven consecutive 2000 cm^−1^ bins. In 2025, we published the ABG-IMRHT room-temperature line list [13] using a “B1b” PES refinement (also generated in 2023) that achieved 0.0055 cm^−1^ accuracy for reliable ^14^N_2_^16^O levels in HITRAN2020 [7], with smaller and more consistent deviations for *J* ≥ 50 compared to the UCL TYM PES. It was a major upgrade over the Ames-1 PES [6]. Compared to the OCS PES refinement accuracies [14] of σ_rms_ = 0.007 cm^−1^ (vs. HITRAN2020 [7]) and 0.009 cm^−1^ (vs. MARVEL [15]), the B1b PES became our 2nd triatomic PES to achieve sub-0.01 cm^−1^ accuracy. In 2025, the UCL group published new series of Comp I-based refinements, including PES53/60/96/60a/96a/60hJ and PES12 in ref. [16], and PES96G/60hJ/60f in ref. [17], reporting accuracies of 0.0049/0.0104 cm^−1^ for PES60hJ (Jacobi/Radau) over 512 *J* = 0/2/5/10/15 levels below 7000 cm^−1^ [17], and 0.0032/0.0028 cm^−1^ for PES60a/96a (Jacobi) over 251/250 *J* = 0/2/5 levels below 7000 cm^−1^ [16]. The latter results were obtained after excluding 28/29 of the 279 available levels from both the PES refinement and the accuracy statistics.

This paper presents our second major upgrade to the N_2_O PES refinement, denoted D2n. The D2n PES achieves 0.001–0.002 cm^−1^ accuracy, as validated against 4808–8462 experiment-based energy levels for all ^14^N_2_^16^O vibrational states below 7000 cm^−1^, i.e., *E*_vib_ = *E*_J_ − 0.4 · *J* · (*J* + 1), with *J*_max_ reaching 88–100. Section 2 presents the δ_calc-expt_ residuals and the *J*-resolved σ_rms_ accuracy of D2n PES energy levels relative to four experiment-based reference sets: RITZ [13,18], NOSL [19], HITRAN2020 [7], and MARVEL [12]. Illustrative comparisons are provided against the Ames B1b PES [13], the UCL TYM PES (2023) [9], and the UCL 2025 PES series [16,17], followed by checks on IR intensities and minor isotopologue accuracy. Section 3 discusses major factors impacting accuracy, the prospects for extending such an accuracy level to higher energies, other isotopologues and molecules, and open questions with our perspectives, e.g., the practical limits of PES refinement within the Born–Oppenheimer approximation framework and the role of nonadiabatic correction and higher order effects. Section 4 summarizes the main findings and briefly outlines future directions.

Efforts are ongoing to further reduce D2n PES deviations at higher energy. This process has taken longer than anticipated, and further work, including the development of a new dipole moment surface, remains to be completed in the next two years. The final version is expected to reach 0.01–0.02 cm^−1^ accuracy for *E*_vib_ > 10,000 cm^−1^ and maintain 0.002 cm^−1^ or better accuracy below *E*_vib_= 7000 cm^−1^. Once complete, it will be reported in a separate publication. In the meantime, an intermediate PES, denoted D2nB, is reported along with D2n, to document the progress made and to support the comparative analyses in Section 2. Like “B1b”, “D2n” and “D2nB” are the internal alphanumeric codes we use to track various refinement series.

Throughout this work, N_2_O isotopologues are labeled using the unit number of isotope mass integers, e.g., 446 for ^14^N^14^N^16^O (the primary isotopologue), 456 for ^14^N^15^N^16^O, 448 for ^14^N^14^N^18^O, and 458 for ^14^N^15^N^18^O.

## 2. Results

A consolidated technical background is provided in Appendix A.1, Appendix A.2, Appendix A.3, Appendix A.4 and Appendix A.5, beginning with an overview of recent Comp-I PES-based N_2_O PES developments and the differing refinement strategies adopted by the Ames and UCL groups (Appendix A.1). Then it describes the purification and composition of the experimental reference datasets, the weighting scheme, and the iterative coefficient-adjustment process underlying the final 96-parameter refinement of D2n PES (Appendix A.2). It further explains the rationale and impact for using vibrational energies E_vib_ in statistical analyses, and outlines broader principles for consistent, evidence-based accuracy assessment (Appendix A.3). Because an evaluation of UCL-2025 PES accuracy analyses reveals issues conflicting with our principles, e.g., the selective data exclusions and inaccurate descriptions (Appendix A.4), rigorous reassessment and apples-to-apples comparison are reported below with both *J*-resolved and energy-resolved statistics (Section 2.4). Finally, details are presented for seven unreliable reference energies that we identified with solid evidence before excluding them from statistics, tracing each discrepancy to experimental data errors or assignment issues (Appendix A.5).

The D2n refinement started with the B1b PES coefficients. After trials and steps, it reached a weighted σ_rms_ of 0.00109 cm^−1^ and an unweighted σ_rms_ of 0.00188 cm^−1^ for the 762 levels described in Appendix A.2. The input and output files of the refinement are reported as Supplementary Materials and also available at ZENODO repository. The C_∞V_ global minimum geometry on the D2n PES is *r*_NN_ = 1.1268028 Å, and *r*_NO_ = 1.1855225 Å, with harmonic frequencies of 596.3913 cm^−1^, 1298.5211 cm^−1^, and 2281.9881 cm^−1^. Compared to the B1b PES, the bond-length changes are only −1.7 × 10^−6^ Å and +2.5 × 10^−6^ Å, and the harmonic frequency changes are −0.0041 cm^−1^, −0.0246 cm^−1^, and +0.0442 cm^−1^, respectively, both indicating the convergence of these structural and spectroscopic properties.

### 2.1. Unreliable Reference Energy Confirmed by Robust Analysis

As emphasized in Appendix A.3 and Appendix A.4, only those experiment-based energy values that can be proven unreliable with solid evidence may be excluded from figures or σ_rms_ statistics. This principle is strictly followed in our analyses. All energy levels reported in ref. [16] (Mizus et al. 2025a) and ref. [17] (Mizus et al. 2025b) were initially included, regardless of whether they were reference/experimental energy or computed energy, and regardless of whether they carried an asterisk, “O” or “H”. The reference energies identified as unreliable are analyzed in detail in Appendix A.5 and summarized in Table 1. For transparency, part of the D2n PES accuracy statistics still includes these levels, allowing us to report σ_rms_ values both with and without these levels, and to quantify the magnitude of the resulting σ_rms_ reduction.

**Table 1:** Unreliable reference/experiment-based energy values we identified from those in refs. [16,17], compared to their counterpart in RITZ [6,13], MARVEL [9,12], HITRAN2020 [7], NOSL-296 [19], and Ames B1b [13] and D2n PES-based energies.

^14^N_2_^16^O Levels	21^1^0-2e	21^1^0-2f	04^2^0-20f	04^2^0-30f	04^2^1-30e	21^1^1-1f	19^1^0-2f
ref. [16]-Supp. 12 Tables 1 and 2	3168.3487	3168.3551	n/a	n/a	n/a	n/a	n/a
ref. [16]-Supp. 12 Table 3	3168.3133 *^a^*	3168.3187 *^a^*	2507.7879 *^b^*	2722.2352	4887.8960	n/a	n/a
ref. [17]-Supp. 12 Table 2	3168.3133	3168.3187	n/a	n/a	n/a	5319.9645	6472.0561 *^g^*
MARVEL (UCL-TYM)	3168.31325	3168.31866	2507.79322	2722.23951	4887.89600	5319.96446	6472.05609
RITZ (2023)	3168.34831	3168.35342 *^c^*	2507.80714	2722.25340	4887.91696 *^d^*	5319.99922 *^e^*	6472.05607
RITZ (2024)	3168.34831	3168.34599 *^c^*	2507.80725	2722.25322	4887.91703 *^d^*	5319.99179 *^f^*	6472.05606
HITRAN2020	3168.3487	3168.3551	2507.8077	2722.2531	4887.9163	5319.9986	n/a
NOSL-296 (ABG-IMRHT)	3168.34853	3168.35497	2507.80749	2722.25288	4887.91620	5319.99832	6472.05845
Ames-B1b	3168.34206	3168.34850	2507.80101	2722.24706	4887.91468	5319.99364	6472.05865
D2n (this work)	3168.34800	3168.35443	2507.80844	2722.25354	4887.91676	5319.99804	6472.04906

*^a^* these MARVEL energies were marked as from HITRAN2020 in ref. [16] (Supplement #12, Table 3); *^b^* unclear source for this number, not from MARVEL or HITRAN2020; *^c^* sensitive E_RITZ_ for 2f level, we use 3168.35342 ± 0.0004 (2023) instead of 3168.34599 ± 0.008 (2024); *^d^* uncertainty ~0.003 cm^−1^; *^e^* uncertainty 0.0005 cm^−1^; *^f^* uncertainty 0.008 cm^−1^; *^g^* this MARVEL energy for 2f level was listed among 2e levels in ref. [17].

**Figure 1:**
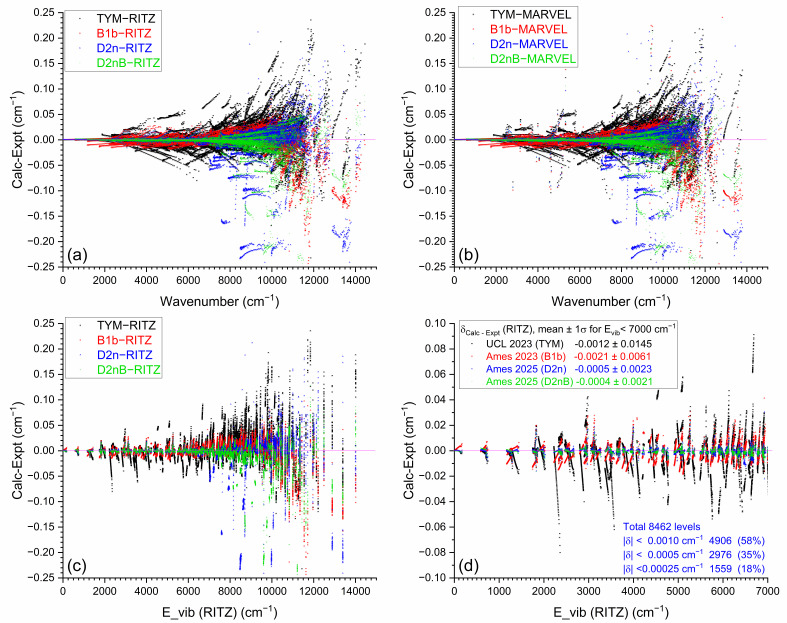
Calc-Expt residuals of ^14^N_2_^16^O rovibrational levels computed using the UCL TYM (black squares), Ames B1b (red circles), D2n (blue triangles), and D2nB (green triangle) PESs, relative to RITZ (in panels (***a***,***c***,***d***)) and MARVEL (in panel (***b***)) collections of experiment-based reference energies. Panels (***a***,***b***) plot residuals against rovibrational energies; panels (***c***,***d***) plot residuals against vibrational energy *E*_vib_ = *E*_J_ − 0.4·*J*·(*J* + 1). Panels (***a***–***c***) show the full energy range, while panel (***d***) focuses on 0–7000 cm^−1^, and reports mean ± σ_rms_ statistics. A magenta line at zero indicates the reference for systematic bias. Note the blue (D2nB) and green (D2n) triangles largely overlap below 8000 cm^−1^. Convergence estimate is better than 0.0001 cm^−1^ for most levels with E_vib_ < 7000 cm^−1^, and within 0.0001–0.001 cm^−1^ for a subset of levels with E_vib_ in the 6000–7000 cm^−1^ range.

**Figure 2:**
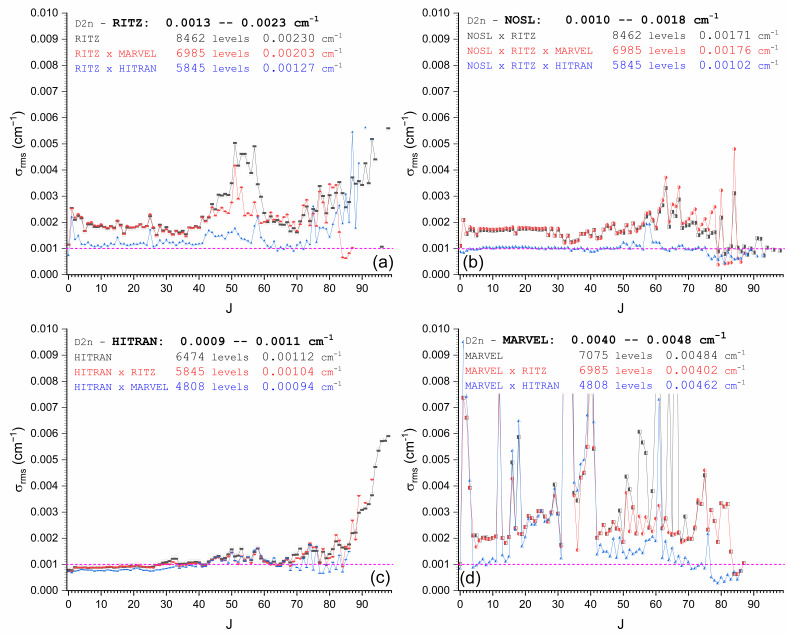
*J*-resolved standard deviations σ_rms_(*J*) between D2n PES-based energies and four reference energy datasets: (**a**) RITZ [13,18]; (**b**) NOSL [13,19] for the same levels as in panel (**a**); (**c**) HITRAN2020 [7]; and (**d**) MARVEL [12]. The smoothness of σ_rms_(*J*) reflects the internal inconsistency of the corresponding reference energy dataset. A magenta line marks 0.001 cm^−1^.

Table 1 contains two sets of E_RITZ_ values. The analyses in Section 2 used the 2024 E_RITZ_ dataset, and the 2024 dataset of 18,943 levels is reported in the Supplementary Materials. Compared to the 2023 set, the 2024 values are nearly identical for most levels; however, certain sensitive levels exhibited shifts approaching 0.01 cm^−1^ and showed noticeably increased uncertainties, e.g., the 21^1^0-2f and 21^1^1-1f levels listed in Table 1. For these two levels, which we have examined in detail, the 2023 values are adopted because they are more reliable and carry significantly smaller uncertainties, as shown in Table 1. The impact on Figure 1 is negligible. Nevertheless, Figure 2 and the statistics in Section 2.3 still use the unmodified 2024 dataset. As a result, the reported mean ± σ_rms_ values relative to E_RITZ_ are slightly larger than they would be if the more reliable values were used, thereby providing even stronger support for the 0.002 cm^−1^ accuracy claim of the D2n PES.

The HITRAN2020 [7] dataset for N_2_O is a compilation of effective Hamiltonian (EH) models fitted to experimental data, including the JPL (Toth) SISAM.N_2_O list [20,21]. Those unreliable energy levels in HITRAN2020 identified in refs. [6,19] have been excluded from the figures and discussions in both ref. [13] and the present work. After the removal of problematic levels, the remaining HITRAN dataset contains 6671 reliable levels, of which 6474 *E*_vib_ < 7000 cm^−1^. Among them, 5846 and 4808 levels have corresponding RITZ and MARVEL energies, respectively.

### 2.2. Compare to UCL-TYM and Ames B1b PES

Figure 1 compares the performance of four potential energy surfaces (PESs)—UCL 2023 [9] (use for the TYM line lists), Ames 2023 B1b [13] (used for the ABG line lists), and the new D2n and D2nB PESs developed in this work—against two independent collections of experiment-based ^14^N_2_^16^O rovibrational energy levels: RITZ [13,18] and MARVEL [12]. All energies and residuals are expressed in cm^−1^. Vibrational energies *E*_vib_ are approximated by subtracting a uniform rotational term 0.40·*J*·(*J* + 1) from the rovibrational energies. The UCL 2025 PES series are not included in the plots because only selected *J* values were reported in refs. [16,17].

The top panels (*a*) and (*b*) show δ_calc–expt_ residuals as a function of rovibrational energy for both RITZ and MARVEL datasets. Relative to the broad scatter of the UCL 2023 PES [9] (black), the B1b PES [13] (red) substantially reduces the spread and removes much of the systematic drift below 10,000 cm^−1^, although noticeable increases in residuals persist at higher wavenumbers. The D2n PES (blue) produces the tightest clustering around zero below 7000 cm^−1^, with deviations confined to a narrow band of only a few thousandths of a wavenumber. The absence of visible curvature or drift indicates improved global consistency. However, at higher energies (7500–11,000 cm^−1^ and near 13,500 cm^−1^), several bands still show deviations larger than those of UCL 2023 PES [9], confirming the need for further refinement. In comparison, the D2nB PES (green) provides an incremental improvement over D2n, reducing the larger deviations across 7000–14,000 cm^−1^, suggesting incremental but meaningful progress. The progression UCL → B1b → D2n (→D2nB) is consistent in both RITZ- and MARVEL-based comparisons, demonstrating that the observed improvements are robust and not sensitive to dataset differences or a few outliers.

Panel (*c*) plots the E_RITZ_-based residuals against *E*_vib_. The sloped patterns in panels (*a*) and (*b*) shrink into vertical lines, making it much easier to track the δ_calc-expt_ behavior of each vibrational state and to visualize the vibrational-state-dependent accuracy. The B1b PES (red) removes much of the scatter structure present in the UCL PES (black), but some notable patterns still remain. The D2n PES (blue) yields a nearly flat distribution up to *E*_vib_ = 7000 cm^−1^, with minimal drift. The D2nB PES (green) brings a modest but clear improvement over D2n, reducing curvature and scatter at higher energies, and effectively targeting the vibrational-dependent errors.

Panel (*d*) expands the *E*_vib_ = 0–7000 cm^−1^ region, and reports the E_RITZ_-based δ statistics for the four PESs. The D2n and D2nB PESs have the smallest mean deviations and σ_rms_ values: −0.0005 ± 0.0023 cm^−1^ for D2n, and −0.0004 ± 0.0021 cm^−1^ for D2nB, indicating minimal systematic bias. Out of 8462 RITZ levels with *E*_vib_ < 7000 cm^−1^, majority of the D2n-based residuals have their magnitude less than 0.001 cm^−1^:

- 4906 levels (58%) with |δ_D2n-RITZ_| < 0.0010 cm^−1^;
- 2976 levels (35%) with |δ_D2n-RITZ_| < 0.0005 cm^−1^;
- 1559 levels (18%) with |δ_D2n-RITZ_| < 0.00025 cm^−1^.

The improvement from D2n to D2nB is modest but it is on the right track, consistent with the trends observed in panels (*a*–*c*). Importantly, D2nB shows no signs of instability or over refinement, reinforcing the reliability of the refinement path.

### 2.3. Reference Data Consistency and J-Dependent Accuracy

The statistical accuracy of a PES refinement may depend critically on the quality and *internal consistency* of the reference dataset. In this paper, internal consistency means that the residuals between experiment-based reference datasets and the assumed exact values remain uniformly small across all relevant *J* values of the included bands. Relative to energy levels variationally computed on highly accurate PES refinements, a reference dataset with high internal consistency should produce smooth, stable residuals, whereas sudden spikes, irregular jumps, or isolated large residuals indicate reduced internal consistency. To assess the impact quantitatively, we compute the *J*-resolved standard deviation σ_rms_(*J*) of δ_D2n_ for levels with *E*_vib_ < 7000 cm^−1^, using four independent sets of experiment-based energies: RITZ [13,18], NOSL-296 (EH-model) [19], HITRAN2020 [7], and MARVEL [12]. For each dataset, we also evaluated the corresponding subsets of levels shared between them. The results are shown in Figure 2, panels (*a*–*d*), together with the number of levels available in each set or subset. Because the full EH-model-based NOSL dataset contains many more levels than have been measured experimentally, panel (*b*) uses NOSL energies only for the same levels included in panel (*a*), enabling a direct comparison between measured energies and the effective Hamiltonian (EH) model fitted to them. Across all panels, the major observation is clear: the apparent statistical accuracy is strongly influenced by the internal consistency and quality of the reference dataset, and this influence can exceed the impact of limitations in the D2n PES refinement.

Because the E_NOSL_ energies are generated from EH-models fitted to the RITZ dataset, they are expected to contain less noise and higher numerical stability along *J*, suggesting higher internal consistency. This is confirmed in panel (*b*), where the σ_rms_(*J*) curves are noticeably smoother than those in panel *a*. Moreover, from panel *a* to panel *b*, the total σ_rms_ decreases by 20–25% when moving from E_RITZ_ to E_NOSL_, dropping from 0.0013–0.0023 cm^−1^ to 0.0010–0.0018 cm^−1^. These results indicate that, *if* a sufficiently large and reliable set of high-resolution experimental data is accurately fitted using a comprehensive EH-model, the resulting EH-based energies for measured levels can be appropriate for PES refinement and accuracy statistics, except for those levels associated with large uncertainties or known issues. On the other hand, the irregularity around *J* = 50–60 in panel (*a*) might reflect localized inconsistencies in the RITZ dataset.

The HITRAN2020-based datasets contain 75–85% as many levels as the datasets in panels (*a*) and (*b*), and not all the E_HITRAN_ values correspond to levels that have been directly measured in lab. This may explain the rising tail of the black curve for *J* ≥ 90 in panel (*c*). Nevertheless, in the *J* = 0–80 range, all three E_HITRAN_-based σ_rms_(*J*) lines lie below 0.002 cm^−1^ and overlap closely, with total σ_rms_ value as small as 0.00094–0.00112 cm^−1^, demonstrating strong internal consistency within the (purified) HITRAN dataset.

Panel (*d*) shows that the E_MARVEL_-based σ_rms_(*J*) lines contain substantially larger values and appear more irregular than those from the other three reference datasets, with total σ_rms_ values of 0.0040–0.0048 cm^−1^, which are roughly 1–4 times higher than those in panels (*a*–*c*). These σ_rms_ indicate limitations of the E_MARVEL_ dataset rather than deficiencies in the D2n PES. Even so, several *J* ranges (e.g., *J* = 4–10, 19–28, 35, 42–54, and 67–88) exhibit reliable or stable σ_rms_ values comparable to those in panel *a*, indicating the E_MARVEL_ values at those *J*s have retained internal consistency, not impacted by data outliers or mixing with low-quality data carrying unreliable uncertainty estimates. At certain *J* values, E_MARVEL_-based σ_rms_ is even smaller than E_RITZ_-based σ_rms_. For example, at *J* = 50, where E_MARVEL_ yields σ_rms_ = 0.00186 cm^−1^ compared to 0.00346 cm^−1^ and 0.00273 cm^−1^ from the two RITZ-based subsets. A similar observation occurs at *J* = 54 (0.00219 cm^−1^ vs. 0.00458 cm^−1^) and *J* = 58 (0.00243 vs. 0.00342 cm^−1^). Given the demonstrated convergence and self-consistency of variational CI calculations across the full *J* range, panel (*d*) simultaneously validates the D2n PES accuracy at these *J* values and highlights unreliable data in E_MARVEL_ dataset.

A second key observation is that the *J*-dependent irregularities occur in different *J* ranges for different reference datasets. If the D2n PES were the limiting factor, a similar pattern of *J*-dependence would appear in all four panels. Instead, each panel or dataset exhibits its own pattern of σ_rms_(*J*) structure, more likely reflecting the internal inconsistency of the dataset. This cross-panel comparison indicates the D2n PES is not the main source of the observed irregularities.

Because the smallest deviations arise from the more internally consistent datasets (E_NOSL_ and E_HITRAN_), one might be tempted to claim a D2n PES accuracy approaching 0.001 cm^−1^. However, E_HITRAN_ contains the fewest vibrational states, and the accuracy of E_NOSL_ for certain levels may be affected by sparse data or by hidden incompleteness and imbalance in the global EH-model, therefore such a claim would be overly optimistic. We deliberately maintain a conservative accuracy estimate of 0.002 cm^−1^, emphasizing robustness across *all* reference datasets rather than relying on the most favorable comparison.

Although the irregular and larger σ_rms_ values in Figure 2 suggest the presence of unreliable data, other possibilities must also be considered. For example, higher vibrational quanta states may not have been included in the PES refinement, or high-resolution data may be overtaken by low-quality measurements with overly optimistic uncertainty estimates. Only case-by-case analysis can reliably identify the true sources of discrepancies.

The NOSL EH-model-based line positions were adopted in the ABG-IMRHT line list and have been incorporated into the HITRAN2024 [22] update. For the 5845 levels in the HITRAN-RITZ subset, the blue line in panel (*b*) and the red line in panel (*c*) are very similar below *J* = 40, whereas the NOSL-based blue line in panel (*b*) remains stable around 0.001 cm^−1^ up to *J* > 90, except at *J* = 57–58. This supports replacing the SISAM.N_2_O line positions used in HITRAN2020 [7] with NOSL-based values, although certain levels at *J* = 57–58 warrant closer examination in future PES refinements.

To the best of our knowledge, this is the first investigation of a polyatomic PES systematically evaluating the consistency of experimental datasets to a sub-0.001 cm^−1^ level.

### 2.4. D2n vs. UCL 2025 PES Series

**Figure 3:**
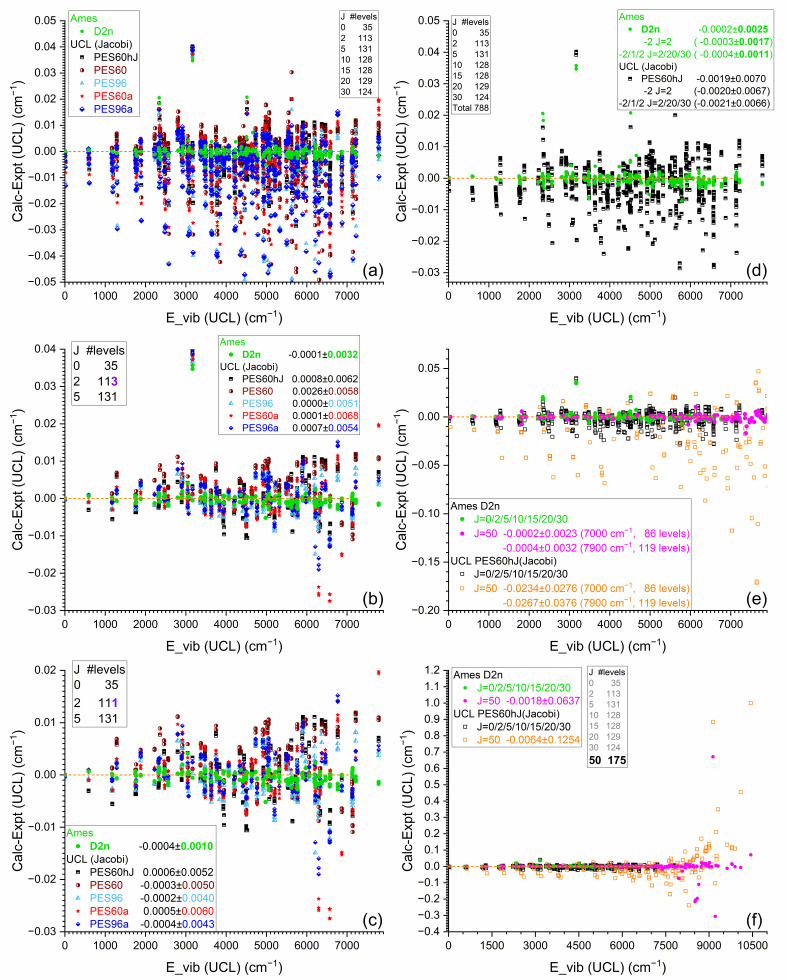
Residuals δ_calc-expt(UCL)_ for ^14^N_2_^16^O levels computed using the D2n PES, compared to the UCL 2025 PES series. All comparisons use the same reference/experiment energy (*E*_J_) set reported in Mizus et al. [16], which is a mix of E_MARVEL_ and E_HITRAN_ and denoted “(UCL)” in panels. Residuals are plotted along vibrational energy *E*_vib_ = *E*_J_ − 0.4·*J*·(*J* + 1). Panels (**a**–**c**) compare D2n results with five UCL PESs. (**a**) Overview of δ for *J* = 0, 2, 5, 10, 15, 20, 30; (**b**) δ distribution and mean ± 1σ for *J* = 0, 2, 5; (**c**) same as panel (**b**), but with the 21^1^0-2e/2f levels removed. Panels (**d**–**f**) compare D2n results with UCL PES96hJ: (**d**) δ and mean ± 1σ for *J* = 0, 2, 5, 10, 15, 20, 30; (**e**) δ and mean ± 1σ at *J* = 50, shown for E_vib_ < 7000 cm^−1^ and <7900 cm^−1^; (**f**) δ and mean ± 1σ at *J* = 50 for all 175 levels with *E*_vib_ < 10,442 cm^−1^.

Figure 3 and Table 2 present an apples-to-apples comparison of δ_calc-expt_ residuals and mean ± 1σ statistics between the D2n PES and the five UCL 2025 PESs. All values are computed using the original reference (and computed) energies extracted from Supplementary file #12 of Mizus et al. [16]. The only distinction is that Figure 3 plots residuals against *E*_vib_ = *E*_J_ − 0.4·*J*·(*J* + 1) to isolate each vibrational states, whereas Table 2 uses *E*_J_ directly for E_max_ cutoffs. This difference does not affect the statistical analysis. In the plots, D2n residuals appear as solid green dots for *J* ≤ 30 in panels (*a*–*f*), and as magenta dots for *J* =50 in panels (*e*) and (*f*).

Among the five UCL PESs, PES60hJ was refined using levels at *J* = 0, 2, 5, 10, and 15, whereas the other four PESs were refined only at *J* = 0, 2, and 5. This fact separates the *J* = 0/2/5 statistics in panels (*b*,*c*) from the statistics up to *J* = 30 and at *J* = 50 in panels (*d*–*f*), particularly for comparisons between D2n and PES60hJ. The labels “60” and “96” denote the total number of coefficients used in the PES and refinement, and the “a” variants (PES60a and PES96a) excluded 28 or 29 levels from their refinement and statistics [16].

All UCL energy levels in ref. [16] were computed in Jacobi coordinates [17]. Our statistics and plots include *all* levels reported in that work [16], including those marked “O” and “H” but except where explicitly noted, e.g., in panels (*c*) and (*d*). To be specific, panel (*c*) excludes the 21^1^0-2e/2f levels, while panel (*d*) reports three sets of statistics: (1) for *all* 788 levels; (2) for 786 levels with 21^1^0-2e/2f levels excluded (“−2 *J* = 2”); and (3) for 783 levels with 21^1^0-2e/2f, 04^2^0-20f/30f, and 04^2^1-30e excluded (“−2/1/2 *J* = 2/20/30”). These exclusions follow the analysis in Appendix A.5 and Table 1.

In panel (*a*), most δ_D2n_ residuals (green dots) are clustered tightly around the δ = 0 line, while the UCL PESs show clear *J*-dependent vertical spreads that grow with energy. This degradation is most pronounced for PES96 (light blue triangles) and PES96a (blue diamonds). PES60hJ (black squares) displays the smallest *J*-dependence among the UCL 2025 PESs (see panel *d*), but the other four UCL PESs show inconsistent accuracy between low and high *J*. All UCL 2025 PESs in panels (*a*) and (*d*) display systematic negative biases.

Panels (*b*) and (*c*) focus on *J* = 0/2/5. The δ_D2n_ residuals form the most compact distribution, with two clear outliers: the 21^1^0-2e and 2f levels. Removing these two levels reduces σ_rms_ (D2n) by two-thirds: from 0.0032 cm^−1^ to 0.0010 cm^−1^. In other words, including these two outliers raises σ_rms_ (D2n) by 220%. By contrast, including the same two levels increase the σ_rms_ of UCL PES by only 13–30% (e.g., 0.0052 → 0.0062 cm^−1^ for PES60hJ; 0.0043 → 0.0054 cm^−1^ for PES96a), because δ(UCL) already contain some residuals with comparable magnitude.

**Table 2:** Statistical mean ± 1σ values of δ_calc-expt_ for the D2n PES (this work) and the UCL 2025 PES series [16,17]. Results are grouped by rovibrational energy cutoff (E_max_ in cm^−1^), maximum rotational quantum number (*J*_max_), and the number of levels (#) included in each dataset. Top section shows the values originally reported in ref. [16] followed by recomputed statistics including *all* levels except 2–5 identified outliers for *J* ≤ 30 (center section) and *J* = 50 (bottom). The number and percentage of levels satisfying |δ| < 0.0010 and |δ| < 0.0005 cm^−1^ are reported for each PES in four sets of statistics. All values are in cm^−1^.

As Originally Reported in Ref. [16], with 279/251/250 Levels (*J* = 0/2/5) or 535 Levels (*J* = 0/2/5/10/15)						
E_max_/*J*_max_/#Levels	PES60hJ	PES60	PES96	PES60a	PES96a	
7000/5/279		0.0054	0.0041			
7000/5/251				0.0032		
7000/5/250					0.0028	
7000/15/535	0.0050					
Recomputed using experiment/UCL levels at *J* = 0/2/5/10/15/20/30 (ref. [16], Supp. file #12, Tables 1–3)						
**E_max_/*J*_max_/#levels**	**PES60hJ**	**PES60**	**PES96**	**PES60a**	**PES96a**	**D2n (This work)**
7795/5/279	0.0008 ± 0.0062	0.0026 ± 0.0058	0.0000 ± 0.0051	0.0001 ± 0.0068	0.0007 ± 0.0054	−0.0001 ± 0.0032
7795/5/277 *^a^* |δ| < 0.0010 cm^−1^ |δ| < 0.0005 cm^−1^	0.0006 ± 0.0052 36 (13%) 17 (6%)	0.0024 ± 0.0050 43 (16%) 21 (8%)	−0.0003 ± 0.0040 51 (18%) 22 (8%)	−0.0002 ± 0.0060 60 (22%) 24 (9%)	0.0004 ± 0.0043 72 (26%) 37 (13%)	−0.0004 ± 0.0010 191 (68%) 107 (38%)
7880/15/533 *^a^* |δ| < 0.0010 cm^−1^ |δ| < 0.0005 cm^−1^	−0.0003 ± 0.0050 73 (14%) 37 (7%)	0.0010 ± 0.0056 100 (19%) 49 (9%)	−0.0022 ± 0.0051 76 (14%) 37 (7%)	−0.0014 ± 0.0063 86 (16%) 38 (7%)	−0.0015 ± 0.0054 103 (19%) 49 (9%)	−0.0004 ± 0.0010 355 (66%) 208 (39%)
8161/30/788	−0.0019 ± 0.0070	−0.0018 ± 0.0102	−0.0064 ± 0.0115	−0.0038 ± 0.0096	−0.0059 ± 0.0120	−0.0002 ± 0.0025
8161/30/786 *^a^*	−0.0020 ± 0.0067	−0.0018 ± 0.0100	−0.0065 ± 0.0113	−0.0039 ± 0.0094	−0.0061 ± 0.0117	−0.0003 ± 0.0017
8161/30/783 *^b^* |δ| < 0.0010 cm^−1^ |δ| < 0.0005 cm^−1^	−0.0021 ± 0.0066 89 (11%) 44 (6%)	−0.0019 ± 0.0100 121 (15%) 59 (8%)	−0.0066 ± 0.0113 84 (11%) 42 (5%)	−0.0040 ± 0.0093 99 (13%) 43 (5%)	−0.0061 ± 0.0117 114 (15%) 54 (7%)	−0.0004 ± 0.0011 523 (66%) 307 (39%)
Recomputed using experiment/UCL levels at *J* = 50 (ref. [16], Supp. file #12, Table 4)						
**E_max_/*J*/#levels**	**PES60hJ**	**PES60**	**PES96**	**PES60a**	**PES96a**	**D2n (This work)**
11,462/50/175	0.0064 ± 0.1254	n/a	n/a	n/a	n/a	−0.0018 ± 0.0637
8900/50/119 *^c^*	−0.0267 ± 0.0376	n/a	n/a	n/a	n/a	−0.0004 ± 0.0032
8000/50/86 *^c^* |δ| < 0.0010 cm^−1^ |δ| < 0.0005 cm^−1^	−0.0234 ± 0.027600	n/a	n/a	n/a	n/a	−0.0002 ± 0.0023 47 (55%) 26 (30%)

*^a^* Two outliers removed: the 21^1^0-2e and 2f. See Table 1 and discussion in Section 2.1 and Appendix A.5; *^b^* three additional outliers removed: 04^2^0-20f, 04^2^0-30f, and 04^2^1-30e. See Table 1 and Appendix A.5; *^c^* for *J* = 50, *E*_J_ = 8900 or 8000 cm^−1^ corresponds roughly to *E*_vib_ = 7900 or 7000 cm^−1^, respectively.

A key observation in panels (*b*) and (*c*) is that most of the 28–29 levels excluded from the PES60a and PES96a refinements are accurately reproduced by the D2n PES-based calculations. These levels should be considered as reliable as the remaining 250–251 levels, at least with uncertainties well within the 1σ spread of the UCL PES series. They should not have been selectively excluded from PES refinements, especially not from refinements restricted to the lowest *J* values. The mean ± 1σ values in panel (*c*) therefore provide a more inclusive and realistic estimate of UCL PES accuracies at *J* = 0/2/5 than the values reported in ref. [16] (see Table 2).

Panel (*d*) compares D2n and PES60hJ for more than 780 levels selected at discrete *J* values up to 30. Removing the 21^1^0-2e/2f outliers reduces the σ_rms_(D2n) by 32% (0.0025 → 0.0017 cm^−1^). Removing three additional outliers at *J* = 20 and 30 (see Table 1 in Section 2.1, and Appendix A.5) further reduces the σ_rms_(D2n) to 0.0011 cm^−1^, consistent with the 0.0010 cm^−1^ value in panel (*c*). Thus, including these five outliers raises σ_rms_(D2n) by 130% (0.0011 → 0.0025 cm^−1^). This demonstrates that the accuracy of D2n PES has made it sensitive to the inconsistency and unreliable data in the reference dataset. Potentially, the σ_rms_(D2n) might be further reduced upon robust δ analysis for additional reference energies, but that is beyond the scope of this work.

For PES60hJ, the total σ_rms_ increase from 0.0052–0.0062 cm^−1^ (panels *b*,*c*) for *J* = 0/2/5 to 0.0066–0.0070 cm^−1^ (panel *d*) for *J* up to 30. The σ_rms_ increases due to outliers is smaller (0.0010 → 0.0004 cm^−1^, or 19% to 6%). The overall scatter remains several times larger than that of δ(D2n), and the σ_rms_ is roughly five times larger than σ_rms_(D2n).

Panels (*e*,*f*) present δ residuals and mean ± 1σ values at *J* = 50, with δ(D2n) shown in magenta and PES60hJ in orange. Lower *J* data are included in the background (green for D2n, black for PES60hJ). Panel (*e*) reports mean ± 1σ values for *E*_vib_ < 7000 cm^−1^ and <7900 cm^−1^. Panel (*f*) includes all 175 levels. For D2n PES, the σ_rms_ increases from 0.0010 cm^−1^ (low *J*) to 0.0023 and 0.0032 cm^−1^ at *J* = 50. The speed of this increase is relatively modest compared to the 3–5× rise in the PES60hJ, whose σ_rms_ grows from 0.0066 cm^−1^ to 0.0276 and 0.0376 cm^−1^. Even in panel (*f*), the σ_rms_(D2n) of 0.064 cm^−1^ remains roughly 50% smaller than the corresponding σ_rms_(PES60hJ), indicating D2n PES reliability at high energy and high *J*.

Across six panels, the mean δ(D2n) values remain consistently close to zero, typically 0.0001–0.0004 cm^−1^, except for the 175 *J* = 50 levels in panel (*f*), where the mean is 0.0018 cm^−1^. Above *J* = 5, the mean δ(D2n) are consistently much smaller than those of the UCL 2025 PES series. The mean δ for PES60/90/60a/90a are listed in Table 2. In extreme cases (panel *e*), the difference exceeds a factor of 100: −0.0002 cm^−1^ vs. −0.0234 cm^−1^ for the 86 *J* = 50 levels with *E*_vib_ < 7000 cm^−1^. For *J* ≤ 30 (panel *d*), the mean δ(PES60hJ) residuals are 4–9 times larger than those of δ(D2n), confirming the systematic bias in D2n PES is substantially smaller.

Table 2 compiles the σ_rms_ values reported by Mizus et al. [16] for the five UCL 2025 PESs, along with their E_max_, *J*_max_ and dataset sizes. The second section of Table 2 reports mean ± 1σ statistics for *J* ≤ 5, 15, and 30. The bottom section reports statistics at *J* = 50. The counts and percentages of |δ| below 0.0010 and 0.0005 cm^−1^ are also listed in four rows. For D2n, 55–68% (majority) of |δ_D2n_| residuals fall below 0.0010 cm^−1^, and 30–39% below 0.0005 cm^−1^, respectively. These percentages are 2.5–7 times those of the UCL PESs. In this aspect, the best UCL performer (PES96a) has only 26% of |δ| below 0.0010 cm^−1^ at *J* ≤ 5, dropping to 19% at *J* ≤ 15 and 15% at *J* ≤ 30. In ref. [16], it did not report *J* = 50 levels for PES60/90/60a/90a, and PES60hJ has no |δ| below 0.0010 cm^−1^ at *J* = 50.

As discussed in Section 2.3, E_MARVEL_-based σ_rms_(*J*) values are sometimes smaller than the E_RITZ_-based values, and *J* = 50 is one such case. Based on our σ_rms_ = 0.0011 cm^−1^ (*J* ≤ 30) and σ_rms_ = 0.0023 cm^−1^ (*J* = 50), the dataset in Mizus et al. [16] likewise supports an accuracy claim of 0.002 cm^−1^ for all states with *E*_vib_ < 7000 cm^−1^ computed on the D2n PES. The mean and σ_rms_ we report in Table 2 for the UCL 2025 PES series are more inclusive and reliable.

**Figure 4:**
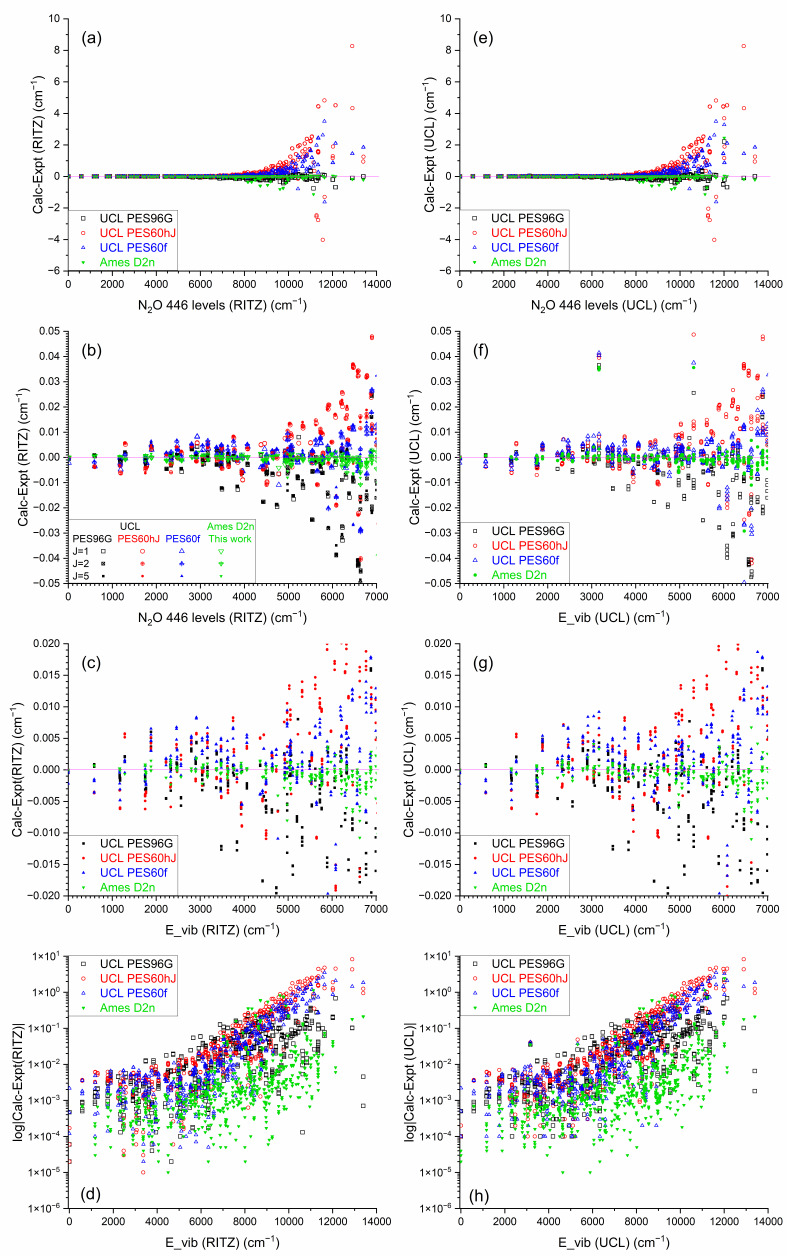
Residual δ*_calc-expt_* for *J* = 1/2/5 levels computed using the D2n PES (this work) and three UCL 2025 PESs: PES96G, PES60hJ and PES60f [17], relative to two reference datasets. Left panels (**a**–**d**): δ against E_RITZ_ values available for 715 out of 756 levels. Right panels (**e**–**h**): δ against the mixed E_MARVEL_ + E_HITRAN2020_ dataset used in ref. [17] (“UCL” reference) for 756 levels. Panels (**a**,**e**): full energy range 0–14,000 cm^−1^; (**b**,**f**) zoomed view of 0–7000 cm^−1^; (**c**,**g**) zoomed view of residuals within ±0.020 cm^−1^; (**d**,**h**) logarithmic overview of log_10_|δ| in full energy range 0–14,000 cm^−1^.

A supplementary file accompanying the Mizus et al. (2025b) [17] provides reference/experimental energies for 756 *J* = 1/2/5 levels up to 13,407 cm^−1^, together with corresponding UCL calculations on PES60hJ, PES60f, and PES96G (all computed in Radau coordinates). Energy levels marked with an asterisk were excluded from the σ_rms_ statistics in that work. These three PESs were presented as more reliable options for line intensity calculations. To re-assess their performance, we re-computed δ and mean ± σ_rms_ using both the original “UCL” dataset (E_MARVEL_ + E_HITRAN2020_) of 756 levels and the E_RITZ_ energies available for the subset of 715 levels. The results are summarized in Figure 4 and in Table 3. Because the rotational contribution at *J* = 5 is only ~10 cm^−1^, using either the rovibrational energy (*E*_J_) or the vibrational energy (*E*_vib_) does not affect the large-scale patterns shown in Figure 4.

**Table 3:** Statistical mean ± 1σ values of δ_calc-expt_ for the D2n and D2nB PESs (this work), compared to UCL PES60hJ, PES60f, and PES96G as reported in ref. [17]. Each statistics lists the maximum rovibrational energies (E_max_ in cm^−1^), maximum rotational quantum number (*J*_max_), and number of levels (#) included. Use all applicable data reported in ref. [17]. Top section shows the σ values originally reported in ref. [17] followed by re-computed mean ± 1σ statistics for the full 756-level dataset, except that four identified outliers are excluded from *J* ≤ 5 and *E*_vib_ < 7000 cm^−1^ statistics (center section), and for the E_RITZ_-based dataset of 715 out of 756 levels (bottom section). The number and percentage of levels satisfying |δ| < 0.0010 and |δ| < 0.0005 cm^−1^ are reported for three sets of statistics. Units are in cm^−1^. Note: “(J)” and “(R)” in top section indicate Jacobi and Radau coordinates used in UCL energy calculations. All values are in cm^−1^.

As Originally Reported in Ref. [17], Not All 756 Levels (*J* = 1/2/5) Were Included in Statistics					
E_max_/*J*_max_/#Levels	PES60hJ	PES60f	PES96G	PES60	
7000/15/512	0.0049 (J) 0.0104 (R)				
7000/5/266		0.0038 (R)		0.0041 (J)	
11,845/5/504			0.0339 (J) 0.0434 (R)		
Mean ± 1σ computed using all 756 reference energies available in ref. [17], *J* = 1/2/5.					
**E_max_/*J*_max_/#levels**	**PES60hJ**	**PES60f**	**PES96G**	**D2n**	**D2nB**
13,407/5/756 |δ| < 0.005 cm^−1^ |δ| < 0.0010 cm^−1^ |δ| < 0.0005 cm^−1^	0.297 ± 0.781 172 (23%) 33 (4%) 23 (3%)	0.178 ± 0.423 253 (33%) 60 (8%) 39 (5%)	−0.038 ± 0.130 208 (28%) 71 (9%) 30 (4%)	−0.024 ± 0.141 508 (67%) 263 (35%) 139 (18%)	−0.0063 ± 0.093 424 (56%) 214 (28%) 108 (14%)
11,642/5/745	0.249 ± 0.627	0.155 ± 0.375	−0.038 ± 0.092	−0.026 ± 0.108	−0.0091 ± 0.0299
9000/5/562	0.050 ± 0.093	0.023 ± 0.050	−0.034 ± 0.053	−0.015 ± 0.064	−0.0037 ± 0.0134
7000/5/330	0.0055 ± 0.0136	0.0016 ± 0.0079	−0.0086 ± 0.0191	−0.0003 ± 0.0041	−0.0000 ± 0.0039
7000/5/326 *^a^* |δ| < 0.0010 cm^−1^ |δ| < 0.0005 cm^−1^	0.0053 ± 0.0130 32 (10%) 23 (7%)	0.0014 ± 0.0065 58 (18%) 37 (11%)	−0.0088 ± 0.0184 71 (22%) 30 (9%)	−0.0006 ± 0.0017 205 (62%) 116 (35%)	−0.0003 ± 0.0016 200 (61%) 97 (30%)
Mean ± 1σ computed using all 715 E_RITZ_ energies available, *J* = 1/2/5					
**E_max_/*J*_max_/#levels**	**PES60hJ**	**PES60f**	**PES96G**	**D2n**	**D2nB**
13,407/5/715	0.290 ± 0.746	0.172 ± 0.401	−0.037 ± 0.105	−0.025 ± 0.101	−0.0073 ± 0.0522
11,642/5/706	0.254 ± 0.625	0.155 ± 0.370	−0.034 ± 0.099	−0.024 ± 0.101	−0.0070 ± 0.0521
9000/5/533	0.052 ± 0.094	0.023 ± 0.051	−0.034 ± 0.052	−0.015 ± 0.065	−0.0040 ± 0.0135
8000/5/390	0.0175 ± 0.0371	0.0054 ± 0.0184	−0.0170 ± 0.0311	−0.0032 ± 0.0151	−0.0009 ± 0.0033
7000/5/307 *^b^* |δ| < 0.0010 cm^−1^ |δ| < 0.0005 cm^−1^	0.0055 ± 0.0134 32 (10%) 27 (9%)	0.0014 ± 0.0068 60 (20%) 39 (13%)	−0.0094 ± 0.0188 69 (22%) 29 (10%)	−0.0006 ± 0.0017 192 (62%) 119 (39%)	−0.0003 ± 0.0015 194 (63%) 111 (36%)

*^a^* Four levels are removed: 21^1^0-2e/2f, 21^1^1-1f, and 19^1^0-2f. The 21^1^1-1f level at 5320 cm^−1^ and 19^1^0-2f level at 6472 cm^−1^ were not included in the refinement or statistics of ref. [16]. See Table 1 in Section 2.1 and Appendix A.5 for details; *^b^* use E_RITZ_ for 21^1^0-2e/2f, and 21^1^1-1f levels. See Table 1 in Section 2.1 and Appendix A.5 for details.

In Figure 4, the left-hand panels show δ residuals computed relative to E_RITZ_, while the right-hand panels use the reference energies extracted from ref. [17]. The two sets of results are broadly similar. Presenting both is essential for validating the analysis and demonstrating that the statistical trends are robust and insensitive to the choice of reference dataset. In the top panels, the green δ_D2n_ dots display the smallest scatter around zero, consistent with earlier observations. It should be noted that Radau coordinate calculations were reported [17] to yield slightly larger δ_UCL_ residuals than the Jacobi coordinate calculations used in earlier work [16]. In the log_10_|δ| scale of the lower panels, most |δ_D2n_| points (green) lie below PES60hJ (red) and PES60f (blue). Below 8000 cm^−1^, δ_D2n_ achieves the smallest mean and σ among all four PESs in both E_RITZ_-based and E_UCL_-based statistics in Table 3. The value of −0.0006 ± 0.0017 cm^−1^ is fully consistent with the results reported earlier in Table 2 and Figure 3.

At the same time, the green |δ_D2n_| dots supersede the black |δ_PES96G_| squares at lower energies, with 62% of |δ_D2n_| falling below 0.0010 cm^−1^. At higher energy, the two surfaces become more comparable. This leads to similar overall mean ± σ_rms_ values for D2n and PES96G in the 9000 cm^−1^ and full-range statistics (Table 3), where δ_D2n_ tends to have smaller mean values while δ_PES96G_ exhibits slightly smaller σ values, e.g., −0.024 ± 0.140 cm^−1^ (D2n) vs. −0.038 ± 0.130 cm^−1^ (PES96G), and −0.015 ± 0.065 cm^−1^ (D2n) vs. −0.034 ± 0.052 cm^−1^ (PES96G).

The reduced accuracy of the D2n PES above 7000 cm^−1^ has been partially remedied in the D2nB PES refinement. In Table 3, the full range mean ± σ_rms_ values are reduced to −0.0063 ± 0.094 cm^−1^ (“UCL”) and −0.0074 ± 0.0519 cm^−1^ (RITZ). The accuracy in the 7000–9000 cm^−1^ range is significantly improved, at least for *J* = 1/2/5, with δ_D2nB_ achieving −0.004 ± 0.014 cm^−1^ up to 9000 cm^−1^. Below 7000 cm^−1^, the performance of D2nB is similar to that of D2n, with more than 60% of |δ_D2nB_| < 0.0010 cm^−1^, and more than 30% of |δ_D2nB_| < 0.0005 cm^−1^. These results confirm that the D2n → D2nB refinement path is effective. They also strongly support the conclusion that most reference energies below (at least) 11,500 cm^−1^ are reliable and should not have been excluded from the original PES96G statistics. Note the δ_D2nB_ values are shown in Figure 1, but not in Figure 4.

It is important to note, however, that the (reference) energy list in the supplementary file of ref. [17] is incomplete. Relative to the original MARVEL level set, 15 *J* = 2e levels are missing between 7276 and 7763 cm^−1^, and 19 *J* = 5e levels are missing between 7235 and 7792 cm^−1^. Additional omissions occur below 7000 cm^−1^ and in other *JPS* blocks, e.g., a *J* = 5f level at 5081.61 cm^−1^. Many of these missing levels have MARVEL-estimated uncertainties of 0.001 cm^−1^ or less. Consequently, the statistics in Table 3 should not be regarded as fully inclusive, including both those quoted from ref. [17] and those computed for *E*_vib_ < 7000 cm^−1^.

### 2.5. Isotopologue Accuracy Check

The D2n PES is an extension of the prior B1b PES, whose comparable accuracy across multiple N_2_O isotopologues was emphasized in ref. [13]. It is therefore essential to evaluate whether the D2n PES preserves such a level of isotopologue consistency for accuracy. To this end, we evaluate the D2n performance for several minor N_2_O isotopologues without introducing any isotope-dependent adjustments. The original ab initio Comp I (and the B1a refit) surfaces were constructed with DBOC contributions specific to the 446 isotopologue. These contributions cannot be transformed to other isotopologues in the absence of the underlying ab initio data, nor can they be refined independently from the rest of the PES. We compute the mean absolute deviation |δ| and the root-mean-square deviation σ_rms_ for both the B1b and D2n PESs relative to the HITRAN2020 and RITZ reference datasets. All results in this subsection thus reflect the unmodified D2n PES optimized for the primary isotopologue 446. The results are summarized in Table 4.

Overall, the average |δ| and σ_rms_ values computed using the D2n PES for minor isotopologues are comparable to those obtained with the B1b PES. Both surfaces yield deviations on the order of 0.005–0.05 cm^−1^. Although an accuracy of 0.01–0.04 cm^−1^ is not ideal, the isotopologue-to-isotopologue consistency remains acceptable for studies that do not require ultra-high spectroscopic accuracy. For the 546, 448, and 447 isotopologues, the B1b → D2n upgrade leads to no significant change in accuracy (for levels available to compare). However, for the 456 and 556 isotopologues, the D2n PES displays 30–60% increases in both the mean residual and σ_rms_, suggesting future optimization of vibrational states involving the center ^15^N atom. This conclusion is reinforced by D2nB statistics relative to E_HITRAN2020_: for several minor isotopologues, the D2nB mean and σ values are 1–10% larger than the corresponding D2n values. For the 446 isotopologue, the D2nB achieves |δ| = 0.0013 cm^−1^ and σ_rms_ = 0.0021 cm^−1^ for *E*_vib_(RITZ) ≤ 7000 cm^−1^, slightly better than the D2n values in Table 4 and consistent with the D2nB statistics in Table 3.

**Table 4:** Average |δ| and σ_rms_ (relative to zero) for N_2_O isotopologue energy levels computed using the B1b and D2n PESs, compared against reliable energies in HITRAN2020 and the RITZ level set. Units are in cm^−1^.

ISO	Number of Levels	B1b PES (Ref. [13]) |δ| Avg σ	D2n PES (This Work) |δ| Avg σ	Reference Set
446	6905 18,943 *^a^* 8462 *^b^*	0.0050 0.0062 0.0099 0.0242 0.0051 0.0065	0.0008 0.0011 0.0256 0.1036 0.0013 0.0023	HITRAN2020 RITZ full RITZ *E*_vib_ ≤ 7000 cm^−1^
456	1769 8590 6877	0.0103 0.0117 0.0175 0.0217 0.0168 0.0202	0.0167 0.0184 0.0302 0.0336 0.0273 0.0292	HITRAN2020 RITZ full RITZ *E*_vib_ ≤ 7000 cm^−1^
546	1952 9334 7598	0.0040 0.0059 0.0091 0.0130 0.0083 0.0114	0.0042 0.0065 0.0110 0.0187 0.0084 0.0112	HITRAN2020 RITZ full RITZ *E*_vib_ ≤ 7000 cm^−1^
448	1894	0.0133 0.0168	0.0109 0.0137	HITRAN2020
447	828	0.0044 0.0055	0.0042 0.0053	HITRAN2020
556	5225 4839	0.0225 0.0263 0.0221 0.0259	0.0314 0.0355 0.0299 0.0329	RITZ full RITZ *E*_vib_ ≤ 7000 cm^−1^

*^a^* |δ| avg/σ = 0.0185/0.0311 cm^−1^ for UCL TYM PES (ref. [9]) and 0.0123/0.0326 cm^−1^ for D2nB; *^b^* |δ| avg/σ = 0.0099/0.0145 cm^−1^ for UCL TYM PES (ref. [9]) and 0.0013/0.0021 cm^−1^ for D2nB.

In 2025, Foltynowicz and co-workers [23] reported precision frequency-comb spectroscopy for ^15^N-bearing isotopologues in the 3300–3550 cm^−1^ region, with line position uncertainties below 0.0001 cm^−1^. Without any empirical correction, the D2n PES reproduces ν_1_ + ν_3_ transitions with the following δ_calc-obs_ deviations:

- 446: δ = −0.0001, −0.0002, −0.0004 cm^−1^ for *P*45e, *P*29e, and *P*17e;
- 456: δ = 0.01100, 0.01100, 0.01104 cm^−1^ for *R*0e, *R*23e, and *R*47e;
- 546: δ = −0.0004, 0.0006, −0.0002 cm^−1^ for *P*2e, *P*24e, and *P*47e;
- 556: δ = 0.0116, 0.0151, 0.0114 cm^−1^ for *P*45e, *P*37e (resonance), and *P*1e.

The 446 and 546 isotopologues show excellent agreement within ±0.0007 cm^−1^, whereas the 456 and 556 isotopologues display deviations exceeding 0.010 cm^−1^. This pattern is fully consistent with the statistical results summarized in Table 4.

### 2.6. IR Intensity Check

Upgrading a line list requires iterative improvements on both the PES and the DMS. Achieving a PES accuracy of 0.002 cm^−1^ is only one step toward producing complete, reliable, and internally consistent IR intensity predictions. Although a more accurate PES does not guarantee improved intensity predictions for every vibrational band, it is critical to evaluate the impact of PES refinements on IR intensities and to identify any large or unexpected changes that might indicate over refinement or instability.

Using the *R*16e-GS transitions as a diagnostic, we computed line intensities using the B1b, D2n, and D2nB PESs in combination with the G10K DMS adopted in the ABG-IMRHT line list. Relative intensity differences Δ% between intensity values S_A_ and S_B_ were defined as Δ% = 50%·(S_A_/S_B_ − S_B_/S_A_). Among the 150 transitions below 8000 cm^−1^ with S_296K_(D2n) > 10^−31^ cm/molecule, 49% of the |Δ%| values for the D2n → D2nB upgrade fall below 0.1%, and 80% of the |Δ%| values for both the B1b → D2n and D2n → D2nB upgrades fall below 1.0%. Only 7–8 weak lines (S_296K_ < 5 × 10^−27^ cm/molecule) exhibit differences exceeding 5%, with a maximum |Δ%| value of approximately 15%.

Table 5 summarizes the intensity changes associated with the B1b → D2n and D2n → D2nB upgrades for selected *R*16e and other transitions. The results indicate that the PES refinements are stable and converged, with no dramatic or systematic changes in IR intensities.

For most medium-to-strong vibrational bands at room temperature, the impact of PES refinement upgrades from B1b to D2nB on IR intensities is negligible. This implies that the deviations previously reported for ν_3_-related overtone bands [6,13] require further investigation. Mizus et al. [17] reported that IR intensity deviations (relative to S_HITRAN2020_) in ref. [16] were significantly reduced when Radau coordinates were used instead of Jacobi coordinates, with agreement surpassing Ames-296K [6] for two-thirds of the HITRAN2020 [7] bands. This was an interesting and unexpected result. Although such coordinate-system effects may not directly apply to our VTET [25] calculations—and the underlying cause of the effects requires further investigation—the report has motivated exploration of other possibilities in future DMS and PES studies. This study focuses primarily on PES accuracy. Based on the results in Table 5, the D2n and D2nB PESs appear stable with respect to IR intensities.

## 3. Discussion

Having established the accuracy of the D2n PES refinement, the more important task is to identify the pre-requisites and conditions needed to extend 0.002 cm^−1^ level accuracy to higher energies, additional isotopologues, other triatomic systems, and ultimately larger molecules, and to explore the fundamental limits of achievable accuracy.

### 3.1. Impacting Factors

The PES accuracy discussed in this work is shaped by several interconnected factors, beginning with the quality of the underlying ab initio PES, and the details of the refinement procedure. The molecular complexity, the rigor of the electronic-structure methods and basis sets, geometry grid and representation, and the inclusion of higher-order corrections also impact the quality of starting PES. It is well recognized that a higher quality ab initio PES may greatly facilitate semi-empirical refinement. The Comp-I PES from Schröder et al. (2015) [8] was far more theoretically advanced over the Ames-0 PES used in Ames-1 refinement [6]. Thus, since 2023 it has become the foundation for all N_2_O PES refinement studies. The one-order of magnitude improvement from the 0.02–0.03 cm^−1^ accuracy of the Ames-1 PES [6] to the 0.002 cm^−1^ accuracy of the D2n PES is largely attributable to the superior quality of Comp I PES. A PES of such caliber may be a prerequisite for achieving similar accuracy in other molecules, particularly when reliability at high energy or near dissociation is required.

A second major factor is the number of PES coefficients included in the refinement. From Ames-1 to B1b/D2n/D2nB, the incorporation of higher order terms has reduced σ_rms_ by at least 30% or more. However, we have not attempted to refine the full set of 680 coefficients in the short-range potential, which would likely require stepwise expansion and tight constraints on variation.

A third factor is the accuracy and *internal consistency* of the reference energy set. This was clearly demonstrated in Figure 2 and the associated discussion. For example, differences in methodology and data sources between the RITZ and MARVEL analyses may introduce hidden uncertainties into accuracy claims based on statistical comparisons. Future refinement efforts may require even higher internal consistency and accuracy in experimentally measured line positions and associated energy levels, as even 1–2% data with lower quality could become the limiting factor. At present, experimental uncertainty of 1 × 10^−4^ cm^−1^ for rovibrational transitions is more than sufficient for a 0.0005–0.0010 cm^−1^ target—unless the PES refinement accuracy can be pushed to 0.0002 cm^−1^, and 1 × 10^−6^ cm^−1^ (or 30 kHz) is adequate for pure rotational transitions.

Another important factor is the PES representation itself, including the choice of coordinates and the polynomial basis. Both the coordinate system and the truncation of the polynomial expansion will influence the fitting and refinement accuracy. In principle, alternative PES representations may enable 0.001 cm^−1^ accuracy (or better) for the same N_2_O reference datasets. At high energies, however, high-order polynomial instabilities or curvatures might be a concern.

The quality of the quantum rovibrational variational CI calculations also matters. It includes the robustness of the kinetic energy operator and the convergence with respect to coordinate choice and CI basis size. Convergence defects at high energy or for sensitive states can compromise statistical results and accuracy claims. In some cases, numerical noise introduced by parallel computation may also affect the final PES accuracy.

These considerations naturally lead to the question of whether a theoretical limit exists for (triatomic) PES accuracy within (or beyond) the Born–Oppenheimer approximation, or whether the limit is effectively set by the accuracy of reference data. At present, reliable answers are not available regarding the interconnection between semi-empirical refinement and the hierarchy of physical corrections: basis set extrapolation, scalar relativistic effects, higher-order electron correlation, and DBOC (all included in Comp-I PES), as well as higher order adiabatic corrections, nonadiabatic effects, Breit interactions, and quantum electrodynamics (QED) contributions, etc. It remains unclear whether all of these effects can be represented accurately within a single coordinate system and polynomial basis, or whether some require term-specific functional forms and separate fits.

The identification and quantification of additional impacting factors will be a long-term effort, and some may be molecule-specific. Nonetheless, the factors outlined above already provide a clear framework for understanding the origins and limits of high-accuracy PES refinement.

### 3.2. Extension to Other Isotopologues, and Open Questions

Using the mass-dependent diagonal Born–Oppenheimer correction (DBOC) as an example, we extend the discussion in Section 3.1 to the question of whether similar or even better accuracy can be achieved for other N_2_O isotopologues.

The DBOC correction is included in Comp I PES as a subset of coefficients, but these coefficients cannot be directly transferred to minor isotopologues without access to the original ab initio data. In principle, one can re-compute and refit the DOBC for each isotopologue, producing slightly different starting PESs tailored to each isotope combination. After refining the D2n PES for the primary isotopologue 446, we have two refinement strategies available for a minor isotopologue such as 448: either repeat the entire refinement procedure starting from the isotopologue-specific PES (with its own DBOC subset), or add the difference between the 446 and 448 DBOC subsets to the D2n PES, evaluate the accuracy, and refine only if necessary. These two approaches may yield almost identical or numerically equivalent results. The main uncertainty lies in the pre-refinement accuracy of the second approach. Nevertheless, for minor isotopologues in HITRAN2020, it is highly plausible to achieve 0.002 cm^−1^ accuracy or better, because high-resolution experimental data coverage is typically less complete for minor isotopologues.

For isotopologues with very limited experimental data (e.g., 557), the incremental-correction approach might be the only viable path. In such cases, the resulting PES accuracy will inevitably rely on the accuracy of ab initio DBOC calculation, and (if necessary) the PES refinement should begin with a very small subset of coefficients to avoid over refinement. If the original DBOC calculation is insufficiently accurate, a third strategy may be possible: derive semi-empirical “effective DBOC” (including other mass-dependent) terms for the N and O nuclei by comparing successful refinements for several more abundant isotopologues (e.g., 456/546/556 and 447/448), and then transfer these terms to the even rarer isotopologues. This approach implicitly assumes the existence of an isotopologue-independent PES that can function as the base of uniform 0.002 cm^−1^ or better accuracy across all isotopologues. Whether such a PES exists, and whether it can be reliably extracted out of the extrapolation from 446, 456, 546, to 556, or from 446 and 447 to 448, remain unknown.

A related question arises from Section 2.5 and Table 4: is it possible to construct a mass-independent PES achieving 0.002 cm^−1^ accuracy simultaneously for two or more isotopologues (e.g., 446 and 546)? If not, what is the highest accuracy that two isotopologues can share? What about three isotopologues? These remain open questions with no definitive answers.

Another conceptual question concerns the role of nonadiabatic corrections. Suppose two sets of energy levels have identical mean deviations and σ_rms_: one computed using a PES refined with explicit nonadabatic corrections in the Hamiltonian, and the other computed using a PES refined without them. Are these two PESs physically equivalent, or is the explicitly nonadiabatic PES inherently more realistic, regardless of numerical accuracy or error cancellation? How far can one push accuracy without nonadiabatic and other higher order effects? This too remains unresolved.

Finally, the most practical open question for our immediate applications is the following: Beyond IR intensity, what additional methods or observables can serve as effective and robust diagnostics for tracking PES quality or verifying convergence? Accuracy at high energy and across isotopologues provides essential boundary checks, but these metrics alone do not guide the direction of future refinement work. Identifying new, sensitive, and reliable indicators of PES quality will be crucial for continued progress.

## 4. Materials and Methods

The general procedure and technical framework for BTRHE type line list development have been summarized in ref. [2]. The first step is to use electronic structure programs to generate high accuracy energies and higher-order corrections on thousands of grid points over the PES region of interest. Extrapolation to the basis set limit may be performed. The most expensive calculations may use reduced grids of hundreds of points. The computed energy series augmented by corrections are fitted to a global PES formula consisting of a local-range PES using a high-order Taylor series expansion at the minimum, a long-range PES including dissociation asymptotes, and a damping function to combine them. Use lower-order expansion fits for correction terms if appropriate. Run quality tests on the global PES series to identify the best PES to start refinement. Add additional grid points to the PES if needed, to ensure it is globally positive and stable at high energy. Then we select several hundreds of experimental energy levels to run PES refinement spanning a wide range of energy and *J*, by adjusting coefficients up to 4th~6th order to obtain 0.01–0.02 cm^−1^ (or better) σ_rms_. It is critical to tightly converge the rovibrational energy levels so that the energy differences and refinements are meaningful.

Finite-field ab initio dipole moments are fit to pseudo point charge models. For example, our N_2_O DMS employ polynomial expansions of the pseudo point charges on the ending N and O nuclei, with the second N atom at the origin. The finite-field dipoles can also be extrapolated to the one-particle basis set limit, with core-valence, scalar relativity, and higher-order correlation effects added as corrections. Because the DMS and PES have different shapes, the DMS grid should be iteratively tested and augmented until adequate coverage is achieved.

The rovibrational calculations are carried out within Born–Oppenheimer approximation framework using analytic basis functions, an exact kinetic energy operator, and accurate matrix elements. Affordable corrections beyond the approximation framework can be included later if necessary. The computations yield rovibrational energy levels and tightly converged wavefunctions. The energy differences among the computed levels give the line position of rovibrational transitions. The associated wavefunctions are then used together with the DMS to compute the IR line intensities of all possible transitions up to J_max_, E′_max_, and E″_max_.

In this work, the ^14^N_2_^16^O rovibrational energy levels and wavefunctions are computed using the VTET program [25] up to *J* = 150 and upper state energy *E*′ ≤ 20,000 cm^−1^ (including zero-point energy). Transition dipole moments and Einstein A_21_ coefficients are computed using the same G10K DMS [13] and the same computational basis and parameters employed in the prior ABG-IMRHT line list [13]. The G10K DMS has a fitting σ_rms_ of 5.2 × 10^−7^ a.u. for 8109 points below 10,000 cm^−1^ [13]. All intensity values are reported at 296 K, unless noted otherwise.

More details of N_2_O PES/DMS construction, PES refinements, and line list calculations can be found in refs. [6,13]. See Appendix A.1 for the timeline of Ames N_2_O PES refinements. See Appendix A.2 for the details of D2n PES refinements. All related datafiles, figures and datasheets are reported together as a Supplementary File and are also available at the ZENODO repository (https://doi.org/10.5281/zenodo.18677392).

## 5. Conclusions

This work presents the first triatomic PES refinement achieving 0.001–0.002 cm^−1^ statistical accuracy against the complete experiment-based energy level collections for ^14^N_2_^16^O with *E*_vib_ < 7000 cm^−1^ and *J*_max_ = 88–100, including RITZ, MARVEL, HITRAN2020, and the matched NOSL-296 (EH-model) datasets. We compared the Comp I PES-based D2n (and D2nB) refinements with the Ames B1b PES [13] and the UCL TYM PES [9] in Figure 1, and with the UCL 2025 PES series [16,17] in Figure 3 and Figure 4. The extensive ab initio calculations and corrections incorporated into the Comp I PES provided a robust theoretical foundation, and arguably a pre-requisite, for enabling overall refinement accuracy better than 0.005 cm^−1^. The energy-resolved and *J*-resolved comparisons with the UCL-2025 PES series (Section 2.4) confirmed that D2n PES consistently exhibits the smallest mean residuals and the smallest scatter below *E*_vib_ = 8000 cm^−1^, as well as the highest fractions of |δ| < 0.0010 cm^−1^ and |δ| < 0.0005 cm^−1^ across all energy windows. The D2n PES also passed both the IR-intensity stability check and the isotopologue/*J*-range consistency check with limitations identified for 456 and 556. The pattern is consistent with recent high-precision spectroscopic measurements. These results provide a comprehensive validation of the D2n PES across rotational, vibrational/energy, and isotopic dimensions, establishing a quantitative baseline for future refinements.

In addition, we have reported detailed *J*-resolved σ_rms_ distributions (Figure 2), illustrating how irregularities in σ correlate with the choice of reference datasets across the full *J* range. Beyond confirming the D2n PES accuracy, these results demonstrate conclusively that the accuracy and internal consistency of the reference energy set can be a deciding factor in relative PES refinements and in statistics-based accuracy claims. These results are also consistent with the cross-comparison analysis we reported for the reference datasets in Huang et al. (2025) [13] and introduced in Appendix A.2 of this paper. We also enlisted the major factors that enable or limit such accuracy, the constraints they impose and the possibility for improving and extending similar accuracy to other isotopologues and molecules. Section 3.1 and Section 3.2 present these considerations and outline several open questions in this largely uncharted domain. One of our long-term goals is to establish a refinement strategy that can be effectively applied to other molecules, with the key pre-requisites and limiting factors clearly identified.

In the near term, a realistic objective is to determine the highest energy range over which 0.002 cm^−1^ accuracy can be retained, especially since the deviations above 10,000 cm^−1^ were partially reduced in the D2nB refinement. Targeting accuracy better than 0.005 cm^−1^ above 10,000 cm^−1^ may require improved experimental data accuracy and consistency in that region, but such data will probably be crucial. It is also scientifically compelling to explore how the *E*_vib_ < 7000 cm^−1^ accuracy can be further reduced to below 0.001 cm^−1^, or even to the 0.0002 cm^−1^ level. Collaboration with high-resolution experimental groups will likely be critical for such advances.

In parallel, it will be important to explore additional approaches for improving the N_2_O DMS *and* reducing the intensity deviations relative to the reliable experiment-based intensities in HITRAN2020. Identifying new diagnostics beyond IR intensity that can track and guide future PES improvements for *E*_vib_ < 7000 cm^−1^ will also be important.

## Figures and Tables

**Table 5:** Relative intensity changes (in %) for selected *R*16e (or specified) transitions resulting from PES upgrades from the B1b PES [13] to D2n and D2nB PESs (this work). Band labels follow NOSL-296 except for 42^0^0 and 50^0^0. Intensities S_296K_ are computed using the D2n PES with the G10K DMS, in cm/molecule at 296 K and 100% abundance.

Band	Position (cm^−1^)	S_296 K_ (D2n) (cm/Molecule)	B1b → D2n	D2n → D2nB
GS	14.24	2.028 × 10^−22^	+0.030%	−0.008%
ν_2_	603.06	1.246 × 10^−20^	+0.0006%	−0.0007%
2ν_2_^0^	1182.65	5.721 × 10^−21^	+0.004%	−0.016%
ν_1_	1298.61	1.748 × 10^−19^	+0.019%	−0.003%
ν_3_	2236.94	1.026 × 10^−18^	−0.012%	+0.001%
2ν_3_	4429.51	1.003 × 10^−21^	+0.087%	−0.061%
3ν_3_	6591.92	2.075 × 10^−23^	−0.092%	−0.016%
42^0^0 *^a^*	6307.67	3.174 × 10^−24^	+0.32%	+0.05%
50^0^0 *^a^*	6385.52	1.422 × 10^−24^	+0.38%	+0.08%
ν_1_ − ν_2_	709.80	3.729 × 10^−23^	−0.066%	−0.022%
ν_1_ − 2ν_2_^0^	130.23	3.332 × 10^−28^	+3.3%	−4.5%
ν_3_ − ν_1_ + ν_2_ *^b^*	342.90	1.376 × 10^−28^	−0.43%	1.11%
ν_2_ + ν_3_-ν_1_ *^c^*	1491.64	3.182 × 10^−26^	−0.17%	−0.057%
3ν_1_ + ν_2_ + ν_3_ *^d^*	6460.94	3.489 × 10^−28^	−1.4%	−0.072%
3ν_1_ + 3ν_2_^1^ + ν_3_ *^d^*	7815.99	1.923 × 10^−28^	+0.51%	−0.21%
ν_1_ + ν_2_ + 3ν_3_ *^d^*	8333.16	2.263 × 10^−27^	−0.31%	+0.035%

*^a^* Intensities are +2.1% and +1.5% higher than NIST measurements in ref. [24] with line position deviations of −0.0003 cm^−1^ and −0.0011 cm^−1^, respectively; *^b^ Q*14f; *^c^ P*25e; *^d^ Q*15e.

## Data Availability

The original contributions presented in this study are included in the article/Supplementary Materials. Since June 2025, the D2n PES and rovibrational energy levels for the six most abundant N_2_O isotopologues (446, 456, 546, 448, 447, and 556) have been publicly available at https://huang.seti.org/N2O/n2o.html (accessed on 31 January 2026). All relevant data files are also provided as Supplementary Materials and archived at the ZENODO repository (https://doi.org/10.5281/zenodo.18677392), including PES, refinements, energy levels, line lists, reference datasets, and statistical outputs. Further inquiries can be directed to the corresponding author.

## Supplementary Materials

The following supporting information can be downloaded at: https://www.mdpi.com/article/10.3390/molecules31111793/s1, README.pdf lists the directory structure and data format of more than 50 datafiles in Supp.files.20260220.zip.

## Abbreviations

The following abbreviations are used in this manuscript:

ABG-IMRHT	Ames B1b PES + G10K DMS—IAO/MARVEL/RITZ/HITRAN2020/Toth data
Ames	NASA Ames Research Center
BTRHE	Best Theory + Reliable High-resolution Experiment
CCSD(T)	Coupled-Cluster Singles and Doubles with perturbative Triples
CI	Configuration Interaction
DBOC	Diagonal Born–Oppenheimer Correction
DMS	Dipole Moment Surface
EH	Effective Hamiltonian
ESA	European Space Agency
ExoMol	Exoplanet Molecular Opacity Database
HITRAN	HIgh-resolution TRANsmission molecular absorption database
JPL	Jet Propulsion Laboratory
MARVEL	Measured Active Rotational–Vibrational Energy Levels
NASA	National Aeronautics and Space Administration
NOSL	Nitrous Oxide Spectroscopic Line list (NOSL-296)
PES	Potential Energy Surface
QED	Quantum ElectroDynamics
SISAM	Spectral Information System for Atmospheric Measurements
TheoReTS	Theoretical Reims–Tomsk Spectra data
TYM	Tennyson, Yurchenko, and Mellor
UCL	University College London
Note: RITZ is not an abbreviation, it is to honor physicist Walther Ritz (1878–1909).	

## Appendix A

### Appendix A.1. Comp-I Based PES Refinements and Ames PES Evolution

The Comp I PES of Schröder et al. [8] incorporated extrapolation to one-particle basis set limit, core-valence correlation, scalar relativistic correction, mass-dependent diagonal Born–Oppenheimer correction (DBOC), and higher order correlation effects beyond CCSD(T). It is the foundation of all recent N_2_O PES refinements, including the UCL 2023 PES (for TYM line list) [9], the Ames B1b PES (for ABG-IMRHT line list) [13], the UCL 2025 PES series [16,17], and the Ames D2n/D2nB PES reported in this work. At Ames, Comp I PES-based energies were generated on the same 3063-point grid of Ames-1 PES [6] construction and then re-fitted to the same 14th-order polynomial representation. This refitted PES was denoted B1a. During the B1a → B1b refinement, 96 out of the 680 PES coefficients were adjusted. However, Huang et al. [13] incorrectly stated that only 35 coefficients up to quartic order had been refined, which is a misleading and inaccurate description. In comparison, the UCL 2023 PES (TYM) [9] converted the Comp I PES [8] into an 8th-order Morse-sin expansion and refined all coefficients using 6563 MARVEL levels selected at 28 *J*s up to *J* = 80 [9]. The UCL 2025 PES series [16,17] took a different approach: refitting the original ab initio energies underlying the Comp I PES using 53 coefficients, then expanding to 60 and 96 coefficients of the same functional form, after that the full coefficient sets were refined against experimental levels at a few *J*s selected from *J* = 0, 1, 2, 5, 10, and 15.

The Ames-1 PES and Ames-296K line lists were generated in 2021 and published in 2023 [6]. The B1b PES (refined in 2023) and the ABG-IMRHT and Ames-2000K line lists (finalized in 2024) were published in 2025 [13]. Building upon the success of the B1b PES, the D2n PES refinement and relevant analyses were completed between February and April 2025, followed by a detailed comparison with the UCL 2025 PES series [16,17] in January 2026.

### Appendix A.2. D2n PES Refinement

A major section of Huang et al. (2025) [13] was devoted to assessing the consistency and error analysis of N_2_O experimental energy levels and deriving more consistent empirical line positions for use in the ABG-IMRHT line list, where “IMRHT” denotes the combined use of IAO effective Hamiltonian (EH) models [18,26,27] (e.g., NOSL-296 [19]), MARVEL [12], RITZ [13], HITRAN2020 [7], and Toth et al. [20,21]. In brief, defects and deficiencies were identified in the MARVEL collection [12] of 17,572 levels. Many issues stem from the mixing of experimental data sources with different accuracy, uncertainty and coverage, while some other defects came from original data errors or occasional mishandling. In contrast, analysis revealed relatively higher internal consistency in the RITZ collection [13] of 18,943 levels, which was curated from 19,892 levels and used in both ref. [13] and present work. For the ABG-IMRHT line list, the NOSL-296 [19] EH-model-based levels were adopted for ^14^N_2_^16^O. In addition, 0th–2nd order corrections were estimated and applied to about 20 vibrational states above 10,000 cm^−1^, based on comparisons across E_NOSL_, E_RITZ_, E_MARVEL_, and/or E_B1b_.

Those purified empirical energy levels used by the ABG-IMGHT line list are adopted as the base of D2n PES refinement. We followed the B1b PES to adjust the 84 coefficients of orders 0–6, plus 12 additional coefficients corresponding to 7–10 quanta of displacements along a bond length or the bond angle. The reference dataset was expanded to 1645 levels selected at *J* = 0, 1, 2, and 5*n* (*n* = 1–19), including both *e* and *f* symmetries. The final D2n refinement used 762 levels, including 54, 75, 33, 80, 46, 83, 46, 83, 45, 82, 71, 42, 13, 6, and 3 levels from the *J*-symmetry blocks 0e, 2e, 2f, 5e, 5f, 10e, 10f, 15e, 15f, 30e, 45e, 60e, 75e, 80e, and 85e, respectively. They include 137, 201, 320, 71, 23, and 10 levels in the energy ranges < 3000 cm^−1,^ 3000–5000 cm^−1^, 5000–7000 cm^−1^, 7000–8000 cm^−1^, 8000–10,000 cm^−1^, and >10,000 cm^−1^, respectively. Of the 762 levels, 722 are normally weighed at 1.0, while 15, 15, 1, 2, 2, 2, 3 levels were weighed at 0.001, 0.01, 0.05, 0.1, 0.2, 0.5, and 10.0, respectively. Above 10,821 cm^−1^, only one level at 12,891.05 cm^−1^ was included with a 0.5 weight. The variation ranges allowed for each coefficient were initially set to ±5%, then relaxed as needed. At the end, 17 of the 96 short-range expansion coefficients changed by more than 20%, with a maximum of 49% for a quartic term. It should be noted that the selection of *J* (and thus the number of levels) and/or the weights of certain levels were adjusted as needed after each refinement step or trial.

### Appendix A.3. Statistical Data ↔ Accuracy Claims

It is more self-consistent to use vibrational state energies, e.g., *E*_vib_ at *J* = 0 or *l*_2_, for defining energy cutoff and computing statistics, instead of the *J*-dependent rovibrational energies. Using *E*_vib_ can isolate each vibrational state cleanly, and the differences between *E*_J_ and *E*_vib_ become more prominent in full-range comparisons. At low *J* (e.g., *J* = 1–5), however, the differences are too small to visualize relative to the energy scale of figures in this paper. For convenience, the vibrational energy is approximated as *E*_vib_ = *E*_J_ − 0.40·*J*·(*J* + 1), where 0.40 cm^−1^ is an effective rotational constant applied uniformly to all states.

Ideally, to ensure that our stated accuracy effectively applies to *all* vibrational states below the specified upper limit, e.g., 7000 cm^−1^ in this work, the effective rotational constant should be larger than 0.419 cm^−1^, the *B*_0_ value of N_2_O 446 ground state. A smaller value may cause a few vibrational states just below 7000 cm^−1^ to appear slightly above the cutoff, effectively lowering the limit slightly. Fortunately, tests show that using *B*_0_ = 0.40 cm^−1^ does not affect the statistical accuracy we report for the D2n PES up to *E*_vib_ = 7000 cm^−1^. Using 0.42 cm^−1^ instead increases the number of RITZ levels below *E*_vib_ = 7000 cm^−1^ by only 1.1% (from 8462 to 8554), and the number of levels with |δ| < 0.0010 cm^−1^ increases by a similar 1.2% (from 4906 to 4967). The total σ_rms_ for D2n vs. RITZ residuals remain unchanged at −0.00045 ± 0.00225 cm^−1^. In comparison the UCL-TYM vs. RITZ residuals increase slightly from −0.0012 ± 0.0145 to −0.0014 ± 0.0148 cm^−1^. The impact on the statistics using ref. [17] data is negligible, because only three levels shift from above to below 8000 cm^−1^. The impact on ref. [16]-based statistics is also minimal, with only one *J* = 50 level shifting from above to below 7000 cm^−1^.

A single σ_rms_ value is insufficient to characterize the quality of a PES refinement. In our view, it should also consider how σ_rms_ evolves along the whole *J* range available for analysis, how rapidly σ_rms_ rise at higher energies, and how σ_rms_ vary across different isotopologues. It may also be informative to examine if σ_rms_ correlates with any vibrational quanta of the states involved. In our prior work, the first two aspects were denoted the *J*-dependence and energy-dependence of σ_rms_, while the latter two were denoted isotopologue consistency and vibrational consistency. Perfect consistency corresponds to no systematic change in δ_calc-expt_ with respect to *J*, energy, isotopologue, or vibrational quanta. Such consistency or dependence analysis can also be applied to relative intensity deviations, Δ% = 50% × (S_A_/S_B_ − S_B_/S_A_), which help reveal hidden deficiencies and guide future improvements.

Researchers may hold different views regarding the accuracy and reliability of various datasets and models, and preferences naturally vary. We respect the limitations and insights identified in both prior and current analyses, unless contradicted by new evidence. For PES refinement, the inclusion or exclusion of specific energy levels and/or values does not always require full justification, because PES refinement is partly empirical and some data must be reserved for testing. However, accuracy statistics used to support explicit accuracy claims must include all levels up to the stated J and/or energy cutoffs. Unreliable or bad data may be excluded, but *only* when supported by solid evidence, not merely because they are “suspected”. Furthermore, the dataset used to substantiate an accuracy claim should not be identical to the dataset used for PES refinement, unless it already represents the complete dataset. Otherwise, the appropriate statement is that “this accuracy is achieved on the selected subset”, rather than “the accuracy up to a given cutoff is *X*”.

In Huang et al. (2025) [13], Section 3.1.2 (Figure 5) and Section 3.1.3 (Figure 6) quantitatively demonstrated that: (1) E_RITZ_ (446) contains fewer outliers or contaminations than E_MARVEL_ (446), as evidenced by more consistent σ_rms_ across the available *J* range for both RITZ-based and MARVEL-based PES refinements; (2) E_RITZ_ shows substantially better agreement with E_HITRAN_; (3) E_MARVEL_ exhibits more deviations in the range of ±0.05–0.10 cm^−1^ range below 9000 cm^−1^ when compared with E_NOSL_ energies; and (4) “the consistency and accuracy of general E_MARVEL_ data are affected by the mixing of experimental datasets carrying different accuracy, uncertainty and coverage, including those E_MARVEL_ levels properly determined from multiple transitions bearing different accuracy and uncertainty, but no mis-assignment or errors”. These observations and statements were based upon δ_calc-ref_ plots and comparisons across E_NOSL_, E_RITZ_, E_MARVEL_, E_HITRAN_, and the Ames (B1b) and UCL (TYM) energies, utilizing the strength and accuracy of high-quality effective Hamiltonian (EH) models behind E_NOSL_ and E_HITRAN_, and the consistency and convergence of variational CI rovibrational calculations for E_UCL_ and E_Ames_. Because the NOSL-296 EH-model was fitted from the more reliable RITZ dataset, the ABG-IMRHT line lists adopted E_NOSL_ energies for most 446 transitions below 10,000 cm^−1^.

One might argue that the agreement between E_NOSL_ and E_RITZ_ simply reflects the fact that E_NOSL_ energies were fitted from the RITZ dataset, and therefore it does not prove E_RITZ_ or E_NOSL_ is more reliable than E_MARVEL_. More robust evidence is presented in Sections 2.2 and 2.4, where both E_RITZ_ and E_MARVEL_ are used as references, and the exact same reference/experimental energies—taken directly from the Supplementary Files accompanying the UCL 2025 PES series [16,17]—are used to ensure true apples-to-apples comparisons.

### Appendix A.4. Data Issues in UCL 2025 PES Series Studies

Mizus et al. (2025a) [16] marked many levels in Supplement #12 (Tables 3 and 4) with “O” (outlier) or “H” (HITRAN2020 value), and both categories were excluded from their PES accuracy estimates. In Table 2 of Mizus et al. [16], 28–29 levels are labeled “O” for PES60a and PES96a. After removing those levels, the reported σ_rms_ values decreased from 0.0054 cm^−1^ (PES60) and 0.0041 cm^−1^ (PES96) for the full set of 279 *J* = 0/2/5 levels to 0.0032 cm^−1^ (PES60a) and 0.0028 cm^−1^ (PES96a) for the reduced sets of 251 and 250 levels. But only 20 of those levels were consistently marked as “outliers” in both PES60a and PES96a. From our perspective, a 30–40% reduction in σ_rms_ achieved by removing the ~10% largest deviations is neither surprising nor particularly indicative of the strength of the PES refinement. This behavior is typical in PES error statistics: for any experimental level set with sufficient accuracy, the largest deviations naturally cluster in the highest energy or highly excited vibrational states, reflecting limitations or incompleteness in the refined PES or its polynomial coefficients. Such selective removal of the largest deviations does not meaningfully demonstrate the strength of the refinement; it simply reflects the expected behavior of error statistics when the highest-error levels are omitted. More importantly, most of the excluded levels are not genuine “outliers”, as their reference/experimental values are just as accurate and reliable as the levels retained in the σ_rms_ calculations, as demonstrated in Sections 2.2–2.4 of this work. They were excluded solely because they exhibited larger deviations arising from limitations in the PES refinement itself. In other words, this amounts to saying that “the refinement cannot reproduce these levels well,” which highlights the deficiencies in the PES60a and PES96a rather than problems with the data. Furthermore, exclusion of those levels has led to *larger* deviations for several states between 6000 and 7000 cm^−1^ compared with the original PES60 and PES96 results, e.g., the δ for the 42^0^0 state increased from 0.0065/0.0057 cm^−1^ to 0.0240/0.0163 cm^−1^. The 42^0^0←00^0^0 band was one of the two bands measured by the NIST group with <1% uncertainty [24]. Allowing the refinement to become less accurate for such levels in pursuit of a σ_rms_ “approaching experimental accuracy” risks degrading the quality of the associated wavefunctions and intensity predictions. In short, higher accuracy cannot be achieved by excluding valid data or by effectively lowering the energy cutoff. Accuracy claims based on such selective removal have very limited—if any—scientific merit.

In Mizus et al. (2025a) [16], Figure 1 presented the δ residuals for the PES60 *J* = 2/5 levels, and Section.2.4 stated, “The majority of these values are equal to 0.001 cm^−1^, which coincides with typical FTS accuracy.” This description is incorrect, misleading, and entirely inconsistent with the data shown in the Figure 1 of ref. [16]. It also contradicted the reported σ_rms_ (PES60) of 0.0054 cm^−1^ for the full set of 279 *J* = 0/2/5 levels. According to the numerical values provided in ref. [16], only 4/19/27 out of 35/113/131 levels at *J* = 0/2/5 have |δ*_obs-calc_*| ≤ 0.0010 cm^−1^, corresponding to just 11–21%. In total, only 50 out of 279 levels (18%) meet this threshold. An 18% fraction is far from a “majority”, which is conventionally defined as “*more than half*” (Oxford Dictionary). Actually, the δ distribution further confirms this: the *P*0–*P*20 percentiles of δ span −0.0129 to −0.0012 cm^−1^, while the *P*40–*P*100 percentiles range from 0.0011 to 0.0384 cm^−1^. There is no statistical basis for the claim that most values cluster at 0.001 cm^−1^. Nor can this be dismissed as a typographical error, since the authors explicitly compare it to “typical FTS accuracy”. In contrast, Sections 2.2 and 2.4 of this work present a fully compliant example in which the actual majority of δ_calc-ref_ values fall below 0.0010 cm^−1^, with those δ = 0.0010 cm^−1^ excluded.

Mizus et al. (2025b) [17] used asterisks in its Supplement #12 to exclude certain computed energies from the σ_rms_ calculation. However, it is difficult to understand why these exclusions are not applied consistently for the three PES listed in Table 2 (of Supplement #12), i.e., PES96G, PES60hJ, and PES60f. For example, a *J* = 2 level at 5905.4842 cm^−1^ was excluded from the σ_rms_ calculation for PES96G, yet the same level is included in the σ_rms_ calculation for PES60hJ and PES60f. In total, 33 such inconsistencies are identified among *J* = 2 and *J* = 5 levels below 7000 cm^−1^. All the 33 levels have |δ_PES96G-Expt_| > 0.020 cm^−1^, whereas all remaining |δ_PES96G-Expt_| values below 7000 cm^−1^ fall below 0.020 cm^−1^. Therefore, these “O” marks have the effect of artificially lowering the σ_rms_ reported for PES96G, especially in the region below 7000 cm^−1^. Clearly, the reliability or inclusion of an experimental/reference level should be determined solely by its own quality and accuracy, and should not vary from one σ_rms_ calculation to another. Accuracy claims based on such selective exclusions are misleading and unsound.

### Appendix A.5. Analysis for Unreliable Reference Energy Levels

21^1^0 state, 2e and 2f, at 3168.3 cm^−1^: For *J* = 3–60, E_MARVEL_ and E_RITZ_ show similar agreement with E_NOSL_, with most δ values within ±0.0001–0.0002 cm^−1^. At *J* = 1, |δ_RITZ-NOSL_| < 0.001 cm^−1^, and |δ_MARVEL-NOSL_| < 0.004 cm^−1^. However, E_MARVEL_ for 2e and 2f levels are off by 0.035 cm^−1^, whereas E_RITZ_ maintain consistent agreement with E_NOSL_ at 0.0002 cm^−1^ (2e) and 0.0016 cm^−1^ (2f). These discrepancies were traced to the 74KrSa.602/615 lines [28] used in the MARVEL analysis [12]: the reported wavenumber at 2574.4854 cm^−1^ for the 21^1^0-01^1^0 *P*3e and *P*3f transitions was lower by ~0.06 cm^−1^, directly producing the unreliable E_MARVEL_.

21^1^1 state, 1f at 5320.0 cm^−1^: For most levels in this state, E_RITZ_ agrees with E_NOSL_ to within ~0.001 cm^−1^ or better, and E_MARVEL_ shows comparable or slightly better agreement. However, two E_MARVEL_ values for the 1f and 3f levels deviate by 0.035 cm^−1^. In the MARVEL network, these two levels were connected solely through the 2f level of the 21^1^0 state, which is exactly the level affected by the 0.035 cm^−1^ error described above. Thus, the E_MARVEL_ error in the 21^1^1-2f level propagated directly into the 21^1^1-1f and 3f levels.

04^2^1 state, 30e at 4887.9 cm^−1^: The E_MARVEL_ values for 04^2^1 state exhibit the largest δ discrepancies below 6000 cm^−1^ when compared with E_NOSL_, E_RITZ_, E_HITRAN,_ and E_Ames-B1b_ energies. This issue, previously illustrated in the Figure 6b (and discussed in Section 3.1.3) of ref. [13], was traced to an omitted e/f splitting. Specifically, the *R*29f transition of the 04^2^1-04^2^0 band at 2190.729 cm^−1^ was doubly assigned [29] to both *R*29e (01BaVe.3055) and *R*29f (01BaVe.3111). The expected *e*/*f* splitting is 0.0195 cm^−1^, which explains the E_MARVEL_ deviation.

04^2^0 state, 20f at 2507.8 cm^−1^ and 30f at 2722.2 cm^−1^: For this state, E_RITZ_ show consistent agreement with E_NOSL_ for both e and f components, typically within 0.0001–0.001 cm^−1^. The E_MARVEL_ for the *e* levels shows similar consistent agreement. However, the E_MARVEL_ for the f levels display inconsistent deviations between *J* = 13 and 33, including oscillatory even-*J*/odd-*J* behavior in δ_MARVEL-NOSL_. The δ_MARVEL_ deviation at 30f is traced to the *R*26e transition of the 04^2^3-04^2^0 band at 6430.708 cm^−1^ (07LiKaMaRo.2917) [12], which was also doubly assigned to *R*26f. In the supplemental .docx file of ref. [30] (pp. 53–54), all f-branch transitions of this band were reported with identical wavenumbers to their e-branch counterparts. Because the transition carried a smaller uncertainty, it was more heavily weighted in the MARVEL analysis than the other two transitions. The δ_MARVEL_ deviation at the 20f level is also related to the *R*22f transition (07LiKaMaRo.2914) in ref. [30], for which both NOSL-296 [19] and D2n line lists predict an e/f split of 0.011 cm^−1^. Note the energy value quoted for the 20f level in ref. [16] (Supp.#12, Table 3) leads to an even larger deviation, but that value does not match either E_HITRAN_ or E_MARVEL_ (see Table 1 of this work).

19^1^0 state, 2f at 6472.06 cm^−1^: This state has a 2f level in both E_MARVEL_ and E_RITZ_, but no corresponding 2e level. In ref. [17] (Supplement #12), the E_MARVEL_ for the 2f level was accidentally listed among the 2e levels, introducing an artificial deviation of ~0.02 cm^−1^ due to the e/f splitting. The underlying energy is correct, but the 2f level must be omitted from comparisons, because the PES96G/60hJ/60f-based energies in ref. [17] might correspond to the 2e level, not to the 2f level.
