# Supplementary material for: A 0.002 cm−1-Accurate PES for 14N216O
Source: Molecules. 2026 May 23;31(11):1793. doi: 10.3390/molecules31111793 (PMC13257640; doi:10.3390/molecules31111793)
Supplement: Supplementary file 1 [file molecules-31-01793-s001.zip › README.pdf]

Supplementary Data Files for

A 0.002 cm^{-1}-accurate PES for $^{14}N_2{}^{16}O$

Xinchuan Huang, David. W. Schwenke

1. Compressed File Name:"Supp.files.20260220.zip" or "Supp.files.20260220.tar.xz"

2. Units: cm^{-1} for E', E", wavenumber, Energy, unc, E_xx, diff
   cm^{-1}/molecule.cm^{-2} for S_{296K}); s^{-1} for A_{21}

3. File Structure, Data format, and Notes

```
|    D2n_PES_related_analysis.revised.opju
|                ORIGIN project file, including data and figures, open/extract using Origin Viewer
+---D2n
|    +---Linelist
|    |        n2o.446.D2n-G10K.296K.1E-31.dat.B60K.xz
|    |            iso wavenumber S296K A21 E" (v1v2v3)' (v1v2v3)" (J/P/S/ir)' (J/P/S/ir)" J' J" Parity
|    +---PES
|    |        n2opes2.coeff.dat.D2n              input file required by n2opes2.f90
|    |        n2opes2.f90                        same subroutine as reported along with B1b PES
|    |
|    +---refinement                              [refinement records, for reference only]
|    |        fort.2.try6IMRHT.D2n.vib7K         input file
|    |        fort.4.try6IMRHT.D2n.vib7K         input file
|    |        newfort4.try6IMRHT.D2n.vib7K       updated input file for next run
|    |        out.try6IMRHT.D2n.vib7K.xz         output file
|    |
|    \---Rovibrational.Energy.Levels             v1v2l2v3 for reference only, J+isym even/odd→e/f
|             n2o.446.levels.D2n-B60K.dat.xz     J isym #root Energy, v1v2l2v3, Eref, coeff
|             n2o.447.levels.D2n-B60K.dat.xz     J isym #root Energy, v1v2l2v3, Eref, coeff
|             n2o.448.levels.D2n-B60K.dat.xz     J isym #root Energy, v1v2l2v3, Eref, coeff
|             n2o.456.levels.D2n-B60K.dat.xz     J isym #root Energy, v1v2l2v3, Eref, coeff
|             n2o.546.levels.D2n-B60K.dat.xz     J isym #root Energy, v1v2l2v3, Eref, coeff
|             n2o.556.levels.D2n-B60K.dat.xz     J isym #root Energy, v1v2l2v3, Eref, coeff
|
+---D2nB
|    +---Linelist
|    |        n2o.446.D2nB-G10K.296K.1E-31.dat.xz
|    |            iso wavenumber S296K A21 E" (v1v2v3)' (v1v2v3)" (J/P/S/ir)' (J/P/S/ir)" J' J" Parity
|    +---PES
|    |        n2opes2.coeff.dat.D2nB             input file required by n2opes2.f90
|    |        n2opes2.f90                        same subroutine as reported along with B1b PES
|    |
|    +---refinement                              [refinement records, for reference only]
|    |        fort.2.D2n-nosl.rn-vib7K-J025B     input file
|    |        fort.4.D2n-nosl.rn-vib7K-J025B     input file
|    |        newfort4.D2n-nosl.rn-vib7K-J025B   updated input file for next run
```

| | out.D2n-nosl.rn-vib7K-J025B.xz   output file
| |
| \---Rovibrational.Energy.Levels $v_1v_2l_2v_3$ for reference only, *J*+isym = even/odd→*e*/*f*
| n2o.446.levels.D2nB-B60K.dat.xz   *J* isym #root Energy, $v_1v_2l_2v_3$, E_{ref}, coeff
| n2o.446.levels.D2nB-B90K.dat.xz   *J* isym #root Energy, $v_1v_2l_2v_3$, E_{ref}, coeff
|
\---reference.and.statistics
     +---get_rms_statistics
     | | 20260213.for.446.tar.xz   File collection for $^{14}N_2{}^{16}O$ statistics
     | | B1b/D2n/D2nB/TYM.vs.RITZ/MARVEL/HITRAN…… format given at the top of the file
     | | getrms.f90.xxx and out.xxx   for records only, no format is needed
     | \---minor.isotopologues   reference energy levels and comparison
     | +---447
     | | fort.1903.s11.447.D2n.HITRAN2020   format given at the top of the file
     | | HITRAN2020.447.levels   *J*, isym, Energy, $v_1v_2l_2v_3$, label
     | |
     | +---448
     | | fort.1903.s11.448.D2n.HITRAN2020   format given at the top of the file
     | | HITRAN2020.448.levels   *J*, isym, Energy, $v_1v_2l_2v_3$, label
     | |
     | +---456
     | | fort.1903.s11.456.D2n.HITRAN2020_full   format given at the top of the file
     | | fort.1903.s11.456.D2n.RITZ_full   format given at the top of the file
     | | fort.1903.s11.456.D2n.RITZ_vib7K   format given at the top of the file
     | | HITRAN2020.456.levels   *J*, isym, Energy, $v_1v_2l_2v_3$, label
     | | RITZ.456.levels.v2   *J*, isym, Energy, $v_1v_2l_2v_3$, ief, e/f, unc, #lines
     | |
     | +---546
     | | fort.1903.s11.546.D2n.HITRAN2020   format given at the top of the file
     | | fort.1903.s11.546.D2n.RITZ_full   format given at the top of the file
     | | fort.1903.s11.546.D2n.RITZ_vib7K   format given at the top of the file
     | | HITRAN2020.546.levels   *J*, isym, Energy, $v_1v_2l_2v_3$, label
     | | RITZ.546.levels.v2   *J*, isym, Energy, $v_1v_2l_2v_3$, ief, e/f, unc, #lines
     | |
     | \---556
     | fort.1903.s11.556.D2n.RITZ_full   format given at the top of the file
     | fort.1903.s11.556.D2n.RITZ_vib7K   format given at the top of the file
     | RITZ.556.levels.v2   *J*, isym, Energy, $v_1v_2l_2v_3$, ief, e/f, unc, #lines
     |
     \---reference.datasets
               For.B1b_n2o.446.levels.B1b-B60K.dat.xz   *J*, isym, #root, Energy, $v_1v_2l_2v_3$, E_{ref}, coeff
               For.HITRAN__B1b-HITRAN   format given at the top of the file
               For.MARVEL__marvel.17548.levels.in.UCL.list.TYM format given at the top of the file
               For.RITZ__fort.1322.from.checkritz.v3b.xz   format given at the top of the file
               For.TYM__fort.2011.s125.ucl.TYM.xz   *J*, isym, E_{calc}, E_{TYM}=E_{MARVEL} (Ma) or E_{calc} (Ca)
               energy_levels.ritz   source file of RITZ levels, format given in the file
